# When to Start Population-Wide Screening for Chronic Kidney Disease: A Cost-Effectiveness Analysis

**DOI:** 10.1001/jamahealthforum.2024.3892

**Published:** 2024-11-08

**Authors:** Marika M. Cusick, Rebecca L. Tisdale, Glenn M. Chertow, Douglas K. Owens, Jeremy D. Goldhaber-Fiebert, Joshua A. Salomon

**Affiliations:** 1 Department of Health Policy, Stanford School of Medicine, and Stanford Health Policy, Freeman Spogli Institute for International Studies, Stanford University, Stanford, California; 2 Veterans Affairs Palo Alto Health Care System, Palo Alto, California; 3 Division of Primary Care and Population Health, Department of Medicine, Stanford School of Medicine, Stanford, California; 4 Department of Epidemiology and Population Health, Stanford School of Medicine, Stanford, California; 5 Division of Nephrology, Department of Medicine, Stanford School of Medicine, Stanford, California

## Abstract

**Importance:**

Sodium-glucose cotransporter-2 (SGLT2) inhibitors have changed clinical management of chronic kidney disease (CKD) and made population-wide screening for CKD a viable strategy. Optimal age of screening initiation has yet to be evaluated.

**Objective:**

To compare the clinical benefits, costs, and cost-effectiveness of population-wide CKD screening at different initiation ages and screening frequencies.

**Design, Setting, and Participants:**

This cost-effectiveness study used a previously published decision-analytic Markov cohort model that simulated progression of CKD among US adults from age 35 years and older and was calibrated to population-level data from the National Health and Nutrition Examination Survey (NHANES). Effectiveness of SGLT2 inhibitors was derived from the Dapagliflozin and Prevention of Adverse Outcomes in Chronic Kidney Disease (DAPA-CKD) trial. Mortality, quality-of-life weights, and cost estimates were obtained from published cohort studies, randomized clinical trials, and US Centers for Medicare & Medicaid Services data. Analyses were performed from June 2023 through September 2024.

**Exposures:**

One-time or periodic (every 10 or 5 years) screening for albuminuria, initiated at ages between 35 and 75 years, with and without addition of SGLT2 inhibitors to conventional CKD therapy (angiotensin-converting enzyme inhibitors/angiotensin receptor blockers).

**Main Outcomes and Measures:**

Cumulative incidence of kidney failure requiring kidney replacement therapy (KRT); life years, quality-adjusted life years (QALYs), lifetime health care costs (2024 US currency), and incremental cost-effectiveness ratios discounted at 3% annually.

**Results:**

For those aged 35 years, starting screening at age 55 years, and continuing every 5 years through age 75 years, combined with SGLT2 inhibitors, decreased the cumulative incidence of kidney failure requiring KRT from 2.4% to 1.9%, increased life expectancy by 0.13 years, and cost $128 400 per QALY gained. Although initiation of screening every 5 years at age 35 or 45 years yielded greater gains in population-wide health benefits, these strategies cost more than $200 000 per additional QALY gained. The comparative values of starting screening at different ages were sensitive to the cost and effectiveness of SGLT2 inhibitors; if SGLT2 inhibitor prices drop due to patent expirations, screening at age 55 years continued to be cost-effective even if SGLT2 inhibitor effectiveness were 30% lower than in the base case.

**Conclusions and Relevance:**

This study found that, based on conventional benchmarks for cost-effectiveness in medicine, initiating population-wide CKD screening with SGLT2 inhibitors at age 55 years would be cost-effective.

## Key Points

**Question:**

In the era of sodium-glucose cotransporter-2 (SGLT2) inhibitors, at what age should screening for chronic kidney disease (CKD) begin?

**Findings:**

This cost-effectiveness study compared health benefits, costs, and cost-effectiveness of population-wide CKD screening strategies with varied initiation age and screening frequencies. Screening every 5 years combined with SGLT2 inhibitors from age 55 to 75 years cost $128 400 per quality-adjusted life year (QALY) gained; whereas initiation of screening at age 35 or 45 years produced larger population health benefits, these strategies cost more than $200 000 per QALY gained.

**Meaning:**

Based on conventional cost-effectiveness thresholds, initiation of population-wide CKD screening may be cost-effective at age 55 years.

## Teaser

This cost-effectiveness study compares the clinical benefits, costs, and cost-effectiveness of population-wide chronic kidney disease screening at different initiation ages and screening frequencies.

## Introduction

In 2012, the US Preventive Services Task Force (USPSTF) found insufficient evidence that screening and early detection of chronic kidney disease (CKD) improved clinical outcomes, given limited availability of effective treatment options at that time.^1^ However, sodium-glucose cotransporter-2 (SGLT2) inhibitors have emerged as a practice-changing therapy for CKD, with demonstrated efficacy in improving CKD outcomes and reducing mortality for patients with or without diabetes.^2,3,4^ With growing evidence, clinical guideline organizations, such as Kidney International Improving Global Outcomes (KDIGO), have updated standard of care recommendations for CKD to include SGLT2 inhibitors.^5,6^ The introduction of a new effective treatment has led major guideline groups, including the USPSTF and KDIGO, to revisit population-wide CKD screening recommendations, which could be incorporated into routine wellness visits.^6,7^

In a previous study, we evaluated the potential benefits and costs of population-wide screening for CKD combined with the provision of angiotensin-converting enzyme (ACE) inhibitors or angiotensin receptor blockers (ARBs) plus SGLT2 inhibitors. We found that screening has potential to produce large population health benefits and was cost-effective among US adults aged 35 years or older.^8^ However, our analysis did not evaluate the optimal timing of screening initiation because we limited our analysis to screening strategies that began immediately for different age cohorts, omitting evaluation of strategies deferred to older ages. Because progression to kidney failure occurs over many years to decades, it is possible that screening among younger patients (aged 35-45 years) could be deferred to a later age, yet still capture much of the population benefit from screening and treatment. Careful consideration of the full suite of possible screening initiation ages is important for implementing large-scale population-wide screening. The objective of the current study was to extend our previous work by comparing the cost-effectiveness of population-wide CKD screen-and-treat interventions across screening initiation ages.

## Methods

### Clinical Interventions

For separate cohorts of patients aged 35, 45, 55, 65, or 75 years, we considered intervention strategies that start screening immediately or defer screening to start at older ages. Strategies were further distinguished by different screening frequencies (1 time, every 10 years through age 75 years, or every 5 years through age 75 years).

Screening for CKD consisted of testing for albuminuria (>29 mg/g) using the urine albumin-creatinine ratio (UACR) test and subsequent testing for serum creatinine to calculate estimated glomerular filtration rate (eGFR) accounting for sex and age. Patients with an eGFR lower than 60 mL/min/1.73m^2^ further underwent retroperitoneal ultrasonography to detect structural abnormalities of the kidneys.

For patients with detected CKD, we compared treatment with conventional CKD therapy (ACE inhibitor/ARBs) to therapy that combined ACE inhibitor/ARBs plus SGLT2 inhibitors. Patients who initiated conventional CKD therapy benefited from slowed CKD progression without direct survival benefits.^9,10,11,12^ Those who initiated SGLT2 inhibitors in addition to conventional CKD therapy benefited from slowed CKD progression and reductions in all-cause mortality, as observed in the DAPA-CKD clinical trial.^2,13^ Because we did not explicitly simulate incidence and prevalence of diabetes, we captured the age-specific effectiveness of SGLT2 inhibitors as a weighted average over those with or without diabetes eligible for SGLT2 inhibitor treatment, estimated from the National Health and Nutrition Examination Survey (NHANES).^8,14^

Patients with false-positive results on albuminuria testing who were subsequently treated did not incur benefits from treatment. In rare cases, patients experienced adverse events from conventional CKD therapy and SGLT2 inhibitors, which include angioedema, euglycemic diabetic ketoacidosis, and genital infections.^15,16^ Patients discontinued treatment at rates observed in the DAPA-CKD clinical trial, and all patients discontinued once they developed kidney failure requiring kidney replacement therapy (KRT).

### Natural History Model of CKD

We used our previously published state-transition Markov cohort model to simulate the natural history of CKD over a patient’s lifetime under status quo case finding and treatment with conventional CKD therapy alone.^8^ Model states were defined in terms of eGFR, UACR, as well as CKD detection and treatment status, and the model was evaluated in 3-month time steps.

We classified eGFR stages using the following cutoffs: greater than 90 mL/min/1.73 m^2^ (stage G1), 60-89 mL/min/1.73 m^2^ (stage G2), 45-59 mL/min/1.73 m^2^ (stage G3a), 30-44 mL/min/1.73 m^2^ (stage G3b), 15-29 mL/min/1.73 m^2^ (stage G4), 12-14 mL/min/1.73 m^2^ (kidney failure not necessarily requiring KRT), and lower than 12 mL/min/1.73 m^2^ (kidney failure requiring KRT). Although there is no definitive threshold for initiation of KRT, patients typically initiate between 5 to 15 mL/min/1.73 m^2^, which has been assumed in previous decision-analytic models.^8,17,18^

We classified albuminuria stages using UACR at the following cutoffs: lower than 30 mg/g (no albuminuria), 30 to 299 mg/g (microalbuminuria), and greater than 300 mg/g (macroalbuminuria). We assumed patients do not progress more than 1 eGFR or albuminuria stage within a 3-month interval, and there were no improvements in eGFR and UACR that would result in progressing to a less severe CKD stage.^17,18^ Once CKD was detected, patients remain detected for the duration of their lifetime and are eligible for treatment.

### Data Inputs

Our model was calibrated to provide a good fit to observed age-specific data on CKD prevalence, detection, and treatment status from NHANES, using a bayesian estimation approach described previously (eMethods in Supplement 1).^8,14^

Mortality, costs, and health-related quality-of-life associated with CKD differ by eGFR stages but not by albuminuria stages. Baseline age- and sex-specific mortality rates were obtained from US vital statistics.^19^ We used eGFR-stage–specific mortality hazard ratios (HRs) for those with stage G2 or above derived from the published literature,^20^ assuming patients with kidney failure not requiring KRT had the same mortality rate as patients in stage G4.

Age- and sex-specific baseline health care costs were obtained from the Medical Expenditure Panel Survey.^21^ Patients in stage G3a or worse incurred additional eGFR stage-specific costs obtained from the published literature.^22^ For patients with kidney failure requiring KRT, we used annual per-person cost estimates from the US Renal Data System.^23^ Patients who underwent screening incurred costs from UACR screening and further diagnostic testing (serum creatinine and retroperitoneal ultrasonography) when applicable. We assumed screening was conducted during a routine primary care clinician visit, and therefore we did not include additional clinician costs. Patients who initiated treatment incurred conventional CKD therapy and SGLT2 inhibitor costs obtained from Federal Supply Schedule prices.^24,25^ Patients who experienced adverse events from treatment incurred additional reaction-specific costs.^26,27,28^

We obtained age- and sex-specific health-related quality-of-life weights from the literature and assumed that health-related quality-of-life declined with more advanced eGFR stage.^29,30^ We assumed no differences in health-related quality-of-life weights after patients were detected and/or treated for CKD. Those with treatment-related adverse events incurred health-related quality-of-life decrements for a 3-month interval.^31,32,33^

### Cost-Effectiveness Analyses

#### Base-Case Analysis

The Table describes model parameter base case values and ranges explored in sensitivity analyses. Our cost-effectiveness analysis was designed and conducted according to recommendations from the Second Panel on Cost-Effectiveness in Health and Medicine and the Consolidated Health Economic Evaluation Reporting Standards guideline from the health care sector perspective, which included all formal health care costs (eTable 1 in Supplement 1).^25,44^ We recorded point estimates and 95% uncertainty intervals (UIs)^41^ for all outcomes over the lifetime horizon. Outcomes included cumulative incidence of kidney failure requiring KRT; averted cases of kidney failure requiring KRT; lifetime health care sector costs (2024 USD)^45,46^; life expectancy; quality-adjusted life years (QALYs); and incremental cost-effectiveness ratios (ICERs). Future costs and QALYs were discounted at 3% annually. Costs were adjusted to 2024 USD using Personal Health Care Expenditure and Personal Consumption Expenditure for Healthcare deflators.^45,46,47^ In interpreting ICERs, we referenced a conventional willingness-to-pay benchmark of $150 000 per QALY gained.^48,49,50,51^ In our main results, we reported primarily on strategies for a cohort of patients currently aged 35 years, to focus on the most salient comparison between immediate and deferred screening strategies. Results for other age cohorts are reported in Supplement 1. This study was exempt from institutional review board review because all data were publicly available and it did not include human participants. This study followed the Consolidated Health Economic Evaluation Reporting Standards (CHEERS) reporting guidelines. Analyses were performed from June 2023 through September 2024.

**Table.** Base Case Model Parameters With 95% Uncertainty Intervals (UIs)

Parameters	Value (range)
Screening parameters	
UACR screening sensitivity^34^	0.87 (0.81-0.91)
UACR screening specificity^34^	0.88 (0.84-0.91)
Cost of UACR screening, $^25,35^	52 (40-66)
Probability of treatment initiation after diagnosis^36,37^	0.75 (0.50-1.00)
Diagnosis parameters	
Cost of estimated GFR, $^25,38^	25 (18-31)
Cost of retroperitoneal ultrasonography, $^39^	449 (334-563)
Treatment parameters	
ACEi/ARBs–CKD progression reduction, HR^9,10,11,12^	0.81 (0.52-1.00)
Monthly cost of ACE/ARBs, $^24,25^	36 (27-46)
SGLT2 inhibitors–CKD progression reduction, HR (persons without diabetes)^2,40^	0.51 (0.34-0.72)
SGLT2 inhibitors–all-cause mortality reduction, HR (persons without diabetes)^2,41^	0.54 (0.32-0.86)
SGLT2 inhibitors–CKD progression reduction, HR (persons with diabetes)^2,40^	0.57 (0.45-0.70)
SGLT2 inhibitors–all-cause mortality reduction, HR (persons with diabetes)^2,40^	0.75 (0.56-0.98)
Annual discontinuation rate (SGLT2 inhibitors)^2^	0.051 (0.03-0.08)
Monthly cost of SGLT2 inhibitors, $^24,25^	407 (303-510)
Disutility associated with medication related angioedema adverse event^31^	0.01 (0.003-0.02)
Cost increase from angioedema medication-related adverse event, $^25,26^	4148 (3089-5209)
Proportion of diagnosed persons who experience an angioedema medication-related serious adverse event, %^42^	0.1 (0.01-1.0)
Disutility associated with genital infection adverse event^32^	0.001 (0.0002-0.006)
Cost increase from genital infection adverse event, $^25,27^	161 (119-202)
Annual rate of genital infection adverse event^16^	0.04 (0.03-0.05)
Disutility associated with euglycemic diabetic ketoacidosis adverse event^33^	0.01 (0.005-0.02)
Cost increase from euglycemic diabetic ketoacidosis adverse event, $^25,28^	32 741 (24 413-41 115)
Annual rate of euglycemic diabetic ketoacidosis adverse event^43^	0.002 (0.0002-0.006)
Age-specific diabetes prevalence (among those eligible for SGLT2 inhibitor treatment), %^14^	
30-39 y	11.4 (4.0-21.1)
40-49 y	28.1 (17.6-38.2)
50-59 y	55.5 (47.9-63.0)
60-69 y	40.8 (35.6-46.0)
70-79 y	43.8 (39.6-47.9)
CKD mortality risk parameters, HR	
CKD stage G3a^20^	1.2 (1.1-1.3)
CKD stage G3b^20^	1.8 (1.7-1.9)
CKD stage G4^20^	3.2 (3.0-3.4)
Kidney failure not requiring KRT^20^	3.2 (3.0-3.4)
Kidney failure not requiring KRT^20^	5.9 (5.4-6.4)
CKD quality-of-life adjustments for health states parameters	
CKD stage G2^30,40^	0.85 (0.70-0.96)
CKD stage G3a^30,40^	0.81 (0.66-0.92)
CKD stage G3b^30,40^	0.81 (0.66-0.92)
CKD stage G4^30,40^	0.74 (0.61-0.85)
Kidney failure not requiring KRT^30,40^	0.74 (0.61-0.85)
Kidney failure requiring KRT^30,40^	0.60 (0.51-0.68)
CKD stage-specific monthly added costs, $	
CKD stage G3a for overall population^22,25^	146 (108-182)
CKD stage G3b for overall population^22,25^	394 (293-492)
CKD stage G4 for overall population^22,25^	1144 (843-1441)
Kidney failure not requiring KRT for overall population^22,25^	1144 (843-1441)
Kidney failure requiring KRT for overall population^22,25^	7512 (5582-9442)
Diabetes for overall population (undetected CKD stage G3a)^22,25^	110 (82-139)
Diabetes for overall population (undetected CKD stage G3b)^22,25^	278 (207-349)
Diabetes for overall population (undetected CKD stage G4)^22,25^	643 (479-810)
Diabetes for overall population (undetected kidney failure not requiring KRT)^22,25^	643 (479-810)
Baseline costs, %^21,25^	AHRQ^a^ US expenditure Table (2013 USD converted to 2024 USD) (75-125)

Abbreviations: ACE, angiotensin converting enzyme inhibitors; AHRQ, Agency for Healthcare Research and Quality; ARBs, angiotensin receptor blockers; CKD, chronic kidney disease; GFR, glomerular filtration rate; HR, hazard ratio; KRT, kidney replacement therapy; SGLT2, sodium-glucose co-transporter 2; UACR, urine albumin-creatinine ratio.

^a^ Model diagram and calibrated parameters reported in Cusick et al^8^ Appendix.

#### Sensitivity Analysis

We conducted univariate deterministic sensitivity analyses to assess how changes in model parameters could affect outcomes and strategy comparisons (eMethods, eTable 2 in Supplement 1). In additional scenario analyses, we considered price reductions for SGLT2 inhibitors ranging from 5% to 95% given patent expirations anticipated in 2025.^52^ We also considered a wider range of effectiveness estimates for SGLT2 inhibitors in reference to different efficacy trials.^4^ Finally, we explored more pessimistic implementation scenarios in which the probability of treatment initiation after screening was lower and the rate of treatment discontinuation was higher by up to 25%. We conducted probabilistic sensitivity analyses, summarized using cost-effectiveness acceptability curves and acceptability frontiers, to assess how simultaneous parameter uncertainties changed outcomes and optimal strategies (eMethods, eTable 3 in Supplement 1).^40,53,54^

## Results

### Health Benefits

#### Kidney Failure Requiring KRT

**Figure 1.**
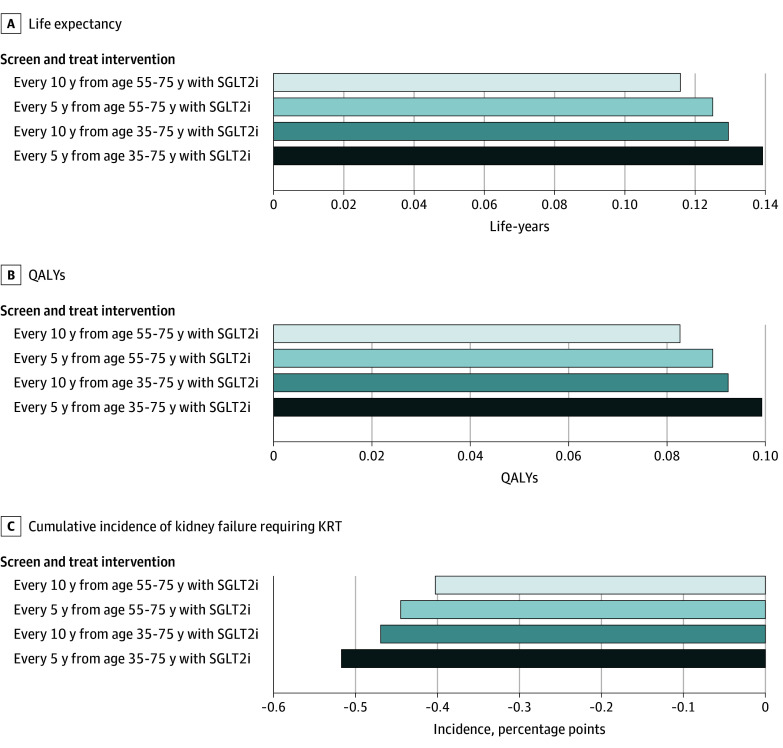
Changes in Outcomes For Population-Wide Screening Interventions^a^ Compared With Status Quo Case Detection and Treatment for Cohort Aged 35 Years A. Increase in discounted life expectancy. B. Increase in discounted quality-adjusted life years (QALYs). C. Reductions in cumulative incidence of kidney failure requiring kidney replacement therapy (KRT). ^a^With SGLT2i indicates the addition of sodium-glucose cotransporter-2 inhibitors to conventional CKD therapy (anigotensin-converting enzyme inhibitors or angiotensin receptor blockers).

Population-wide screening for albuminuria followed by treatment with conventional CKD therapy and SGLT2 inhibitors reduced the burden of kidney failure requiring KRT across all age groups compared with status quo case finding with conventional CKD therapy alone (Figure 1). Under status quo, the cumulative incidence of kidney failure requiring KRT for those aged 35 years was 2.4% (95% UI, 0.7%-5.1%). Under immediate initiation of screening every 5 years at age 35 years, the cumulative incidence was 1.9% (95% UI, 0.5%-4.3%), a reduction of 0.52 percentage points (pp) (95% UI, −1.3 pp to 0.0 pp) compared with the status quo. Deferring the start of screening every 5 years to age 55 years yielded smaller overall benefits than immediate screening, with a reduction of 0.45 pp (95% UI, −1.2 pp to 0.1 pp) compared with the status quo (eTable 4 in Supplement 1).

Among the 158 million US adults currently aged 35 to 75 years, we projected 3.7 million lifetime cases of kidney failure requiring KRT under status quo. Implementing screening every 5 years from age 35 years produced an 18% reduction (662 000 fewer cases of kidney failure requiring KRT; 42 fewer cases per 10 000 persons screened) compared with the status quo, while deferring initiation of screening every 5 years to age 55 years produced a 17% reduction (608 000 fewer cases; 39 fewer cases per 10 000 persons screened) compared with the status quo (eTable 5 in Supplement 1). Screening at age 35 years averted an additional 54 000 cases compared with deferring initiation until age 55 years.

#### Life Years and Quality-Adjusted Life Years

Screening for CKD with conventional CKD therapy plus SGLT2 inhibitors yielded gains in life expectancy and QALYs compared with the status quo across all age groups, with larger benefits observed if screening every 5 years were initiated immediately (Figure 1). Under the status quo, for those aged 35 years, discounted life years and QALYs were 23.8 (95% UI, 23.7-23.9) and 19.1 (95% UI, 17.8-20.1), respectively. Starting screening every 5 years at age 35 years increased life years and QALYs by 0.14 (95% UI, 0.06-0.22) and 0.10 (95% UI, 0.05-0.16) compared with the status quo, respectively. If screening were deferred to start at age 55 years, life years and QALYs increased by 0.13 (95% UI, 0.05-0.20) and 0.09 (95% UI, 0.04-0.14) compared with the status quo, respectively (eTable 6 in Supplement 1).

### Costs and Cost-Effectiveness

Screen-and-treat interventions with SGLT2 inhibitors increased health care costs. For an individual aged 35 years, lifetime health care costs increased on average by $11 900 from $241 100 (95% UI, $182 200-$301 800) to $253 000 (95% UI, $193 300-$314 700) with screening every 5 years starting immediately, compared with the status quo. With screening deferred to start at age 55 years, average health care costs compared with the status quo increased by a smaller increment, $8300, to $249 300 (95% UI, $189 800-$310 700) (eTable 7 in Supplement 1). Immediate initiation of screening was more costly than deferring screening initiation to later ages across age cohorts (eTable 8 in Supplement 1).

**Figure 2.**
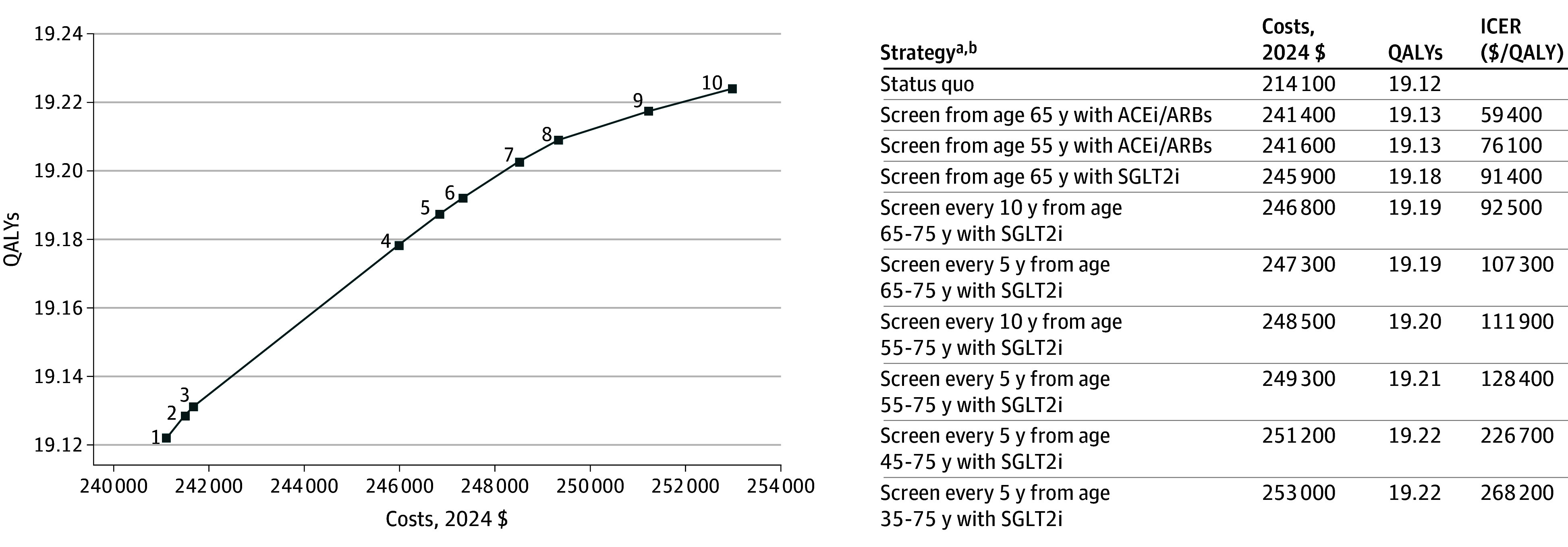
Cost-Effectiveness Plane for Cohort Aged 35 Years ^a^With ACEi/ARBs indicates with conventional chronic kidney disease (CKD) therapy comprising of angiotensin-converting enzyme inhibitors (ACEi) or angiotensin receptor blockers (ARBs). ^b^With SGLT2i indicates with the addition of sodium-glucose cotransporter-2 (SGLT2) inhibitors to conventional CKD therapy.

In terms of cost-effectiveness, for those aged 35 years, initiating screening every 5 years starting at age 65 or 55 years cost $107 300 or $128 400 per QALY gained, respectively, compared with less frequent screening (every 10 years) strategies with the same initiation ages. Starting screening every 5 years at age 45 years or age 35 years increased ICERs to $226 700 or $268 200 per QALY gained, respectively, compared with starting screening 10 years later in each case (Figure 2). For those aged 55 and 65 years, initiating screening every 5 years immediately with SGLT2 inhibitors cost $128 300 and $107 100 per QALY gained compared with corresponding initiation ages with every 10 years frequency (eTable 9 in Supplement 1).

### Sensitivity Analysis

#### Univariate Sensitivity Analysis

**Figure 3.**
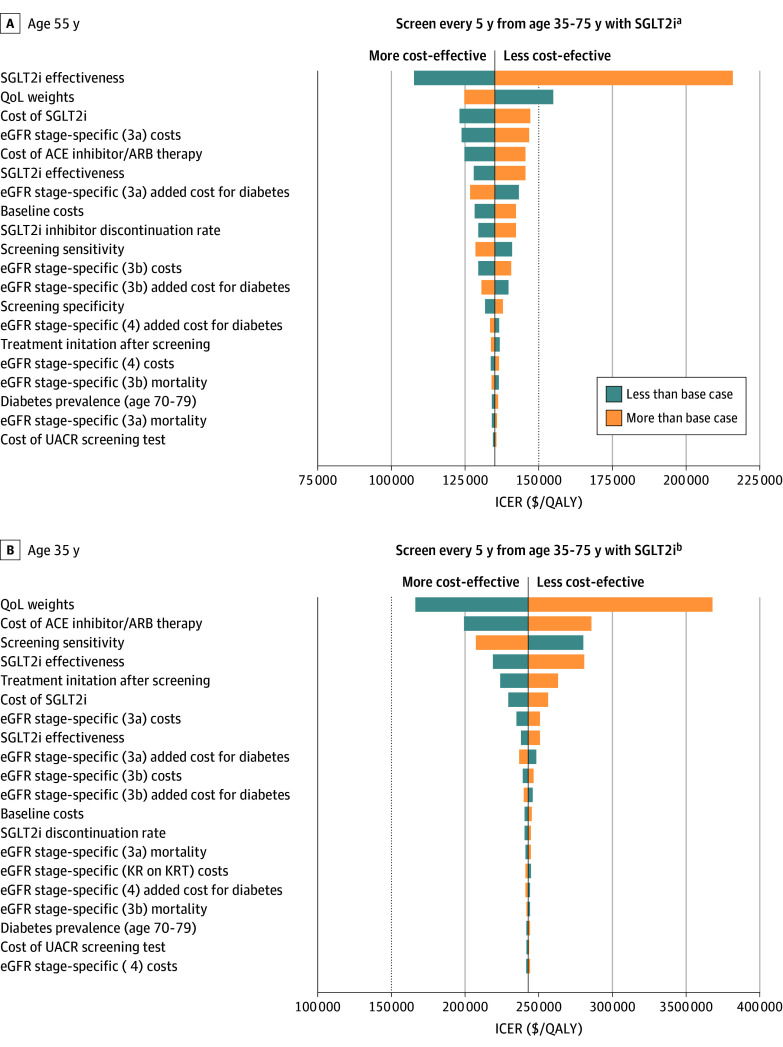
Tornado Plots of Incremental Cost-Effectiveness Ratios (ICERs) of Screening Every 5 Years Combined With Sodium–Glucose Cotransporter-2 Inhibitors (SGLT2i) For Cohort Aged 35 Years A, Initiated at age 55 years. B, Initiated at age 35 years for a cohort with starting age of 35 years. With SGLT2i indicates with the addition of SGLT2is to conventional chronic kidney disease therapy (angiotensin-converting enzyme inhibitors or antigotensin receptor blockers). ACE indicates angiotensin converting enzyme; ARB, angiotensin receptor blocker; eGFR, estimated glomerular filtration rate; QALY, quality-adjusted life years; QoL, quality of life; UACR, urine albumin-creatinine ratio. ^a^Compared with screening every 10 years from age 55 to 75 years with SGLT2i. ^b^Compared with screening every 5 years from age 45 to 75 years with SGLT2i.

Across all patient cohorts, the cost-effectiveness of screening for CKD was most sensitive to changes in SGLT2 inhibitor effectiveness and costs of ACE inhibitors/ARBs and SGLT2 inhibitors (Figure 3; eFigures 1-5 in Supplement 1). For individuals aged 35 years, if SGLT2 inhibitors were less effective at reducing all-cause mortality (HR for those without diabetes, 0.86; HR for those with diabetes, 0.98 vs base-case values of 0.54 and 0.75, respectively), the ICER for screening every 5 years from age 35 years increased to more than $250 000 per QALY gained, and the ICER for waiting to initiate screening at age 55 years increased to more than $200 000 per QALY gained (Figure 3). Results were less sensitive to changes in SGLT2 inhibitor effectiveness in slowing CKD progression. When monthly costs of SGLT2 inhibitors and conventional CKD therapy increased, the ICERs for population-wide screening increased across all cohorts. For interventions initiated at age 45 years or older, results were more sensitive to the cost of SGLT2 inhibitors than to the cost of ACE inhibitors/ARBs (Figure 3).

#### Scenario Analysis

**Figure 4.**
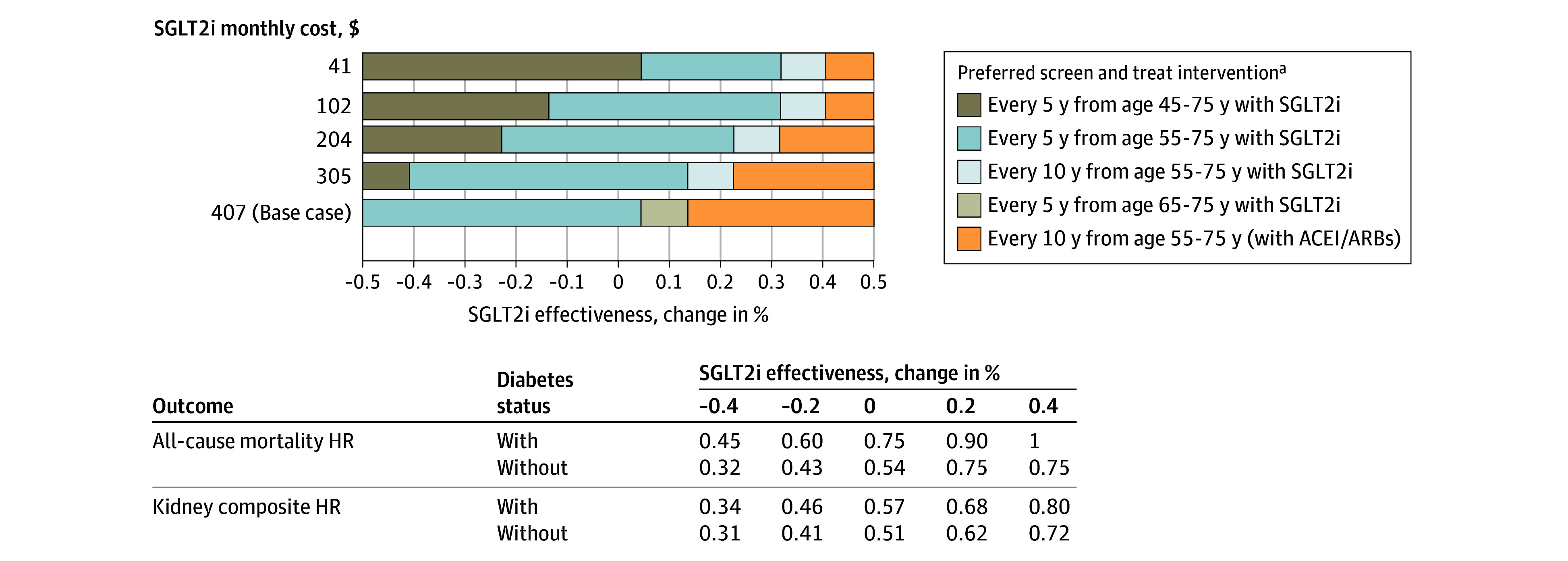
Two-Way Sensitivity Analysis on Sodium-Glucose Cotransporter-2 Inhibitor (SGLT2i) Effectiveness and Monthly SGLT2i Costs at a Willingness-To-Pay Threshold of $150 000 per Quality-Adjusted Life Year (QALY) Gained for Cohort Aged 35 Years HR indicates hazard ratio. ^a^With SGLT2i indicates the addition of sodium-glucose cotransporter-2 inhibitors to conventional chronic kidney disease therapy. With ACEi/ARBs indicates with conventional chronic kidney disease therapy comprising angiotensin-converting enzyme inhibitors (ACEi) or angiotensin receptor blockers (ARBs).

If SGLT2 inhibitor prices decrease, as expected with patent expirations, the cost-effectiveness of CKD screening was more favorable across all age cohorts (Figure 4). For example, with price reductions of 75% ($102 per month), the ICER for immediate initiation of screening every 5 years for those aged 35 years decreased to $203 800 per QALY gained, and the ICER for initiating at age 55 years decreased to $99 900 per QALY gained (eTable 10 in Supplement 1). If prices drop by 90% to $41, the ICER for screening every 5 years starting at age 45 years dropped to $149 800 per QALY gained, whereas immediate initiation at age 35 years remained above $150 000 per QALY gained. Under SGLT2 inhibitor price reductions, selected CKD screening strategies were cost-effective even if SGLT2 inhibitor effectiveness against disease progression and all-cause mortality were lower as reported in other clinical trials (eTable 11 in Supplement 1).^4^ Finally, reducing base-case values for treatment initiation and adherence by 25% to reflect implementation barriers resulted in ICERs for screening every 5 years starting at ages 35 years or 55 years to be $228 400 per QALY gained or $150 800 per QALY gained, respectively (eTable 12 in Supplement 1).

#### Probabilistic Sensitivity Analysis

At a willingness-to-pay threshold of $150 000 per QALY gained, for individuals aged 35 years, screening with SGLT2 inhibitors was preferred in 90% of model runs, among which 70% preferred screening initiation at age 55 years (eTable 13 in Supplement 1).

## Discussion

Demonstration of substantial benefits from SGLT2 inhibitors in reducing kidney disease progression and mortality in patients with CKD has reopened the question of whether screening for CKD is effective and provides good value.^2,3^ In our previous cost-effectiveness analysis,^8^ we found that screening is cost-effective overall for US adults aged 35 years and older, but we did not evaluate when to begin screening different age cohorts, which requires comparison of immediate treatment against strategies that defer screening to older ages. In the present analysis, direct comparison indicated that immediate initiation of screening for patients at younger ages (35 or 45 years) has less favorable cost-effectiveness implications when deferred screening is an alternative. According to conventional cost-effectiveness thresholds, population-wide screening for CKD is cost-effective starting from age 55 years, an important update from our previous recommendation that screening may be cost-effective starting from age 35 years.^8^

Our current analyses showed that immediate initiation of population-wide CKD screening combined with conventional CKD therapy plus SGLT2 inhibitors was associated with the largest population health benefits across US adults aged 35 to 75 years. Compared with the status quo case-finding and treatment with conventional CKD therapy, adding screening every 5 years from age 35 years increased life expectancy and QALYs by at least 0.14 and 0.10 years and reduced cumulative incidence of kidney failure requiring KRT, averting 662 000 cases across the US population. As expected, later initiation of screening provided less benefit than immediate screening. Waiting until age 55 years to initiate screening every 5 years, compared with the status quo, produced life expectancy and QALY gains of at least 0.13 and 0.09, respectively, and averted 608 000 cases of kidney failure requiring KRT. To contextualize these QALY gains against the magnitudes of other screening interventions, universal screening for type 2 diabetes and colorectal cancer produced gains of 0.05 and 0.15 QALYs, respectively, when compared with no screening.^55,56^

Initiating CKD screening at younger ages, for whom CKD prevalence is lower, would also be more costly. Compared with strategies that delay screening every 5 years initiation by 10 years, immediate initiation of screening every 5 years at age 35 or 45 years would cost more than $200 000 per QALY gained, which is greater than commonly used willingness-to-pay thresholds in the US.^48,49,50,51^ These ICERs were higher than our previous estimates of $197 000 and $164 000 per QALY gained,^8^ respectively, when screening every 5 years at age 35 or 45 years was instead compared with less intensive (every 10 years) screening initiated immediately. Waiting until age 55 years to begin screening every 5 years cost less than $150 000 per QALY gained compared with screening every 10 years at age 55 years. The timing of screening initiation has been evaluated and debated in other contexts, such as breast and colorectal cancer screening, highlighting the importance of considering the benefits and harms of screening policies at different ages.^56,57^

The recommended age to initiate CKD screening at age 55 years was somewhat sensitive to SGLT2 inhibitor costs and effectiveness in slowing CKD progression and reducing all-cause mortality, although our results were robust to variation in values for most model parameters examined. If SGLT2 inhibitor effectiveness across both end points were simultaneously reduced by 30%, deferring to screening every 5 years to begin even later, at age 65 years, may be preferred. However, if monthly prices of SGLT2 inhibitors drop by 75%, smaller than average price drops from patent expirations,^52^ screening initiation at age 55 years remains preferred even given lower SGLT2 inhibitor effectiveness. Screening initiation at age 55 years was robust across tested scenarios including clinical implementation challenges from reduced treatment adherence and lower SGLT2 inhibitor effectiveness as reported in other clinical trials.^3,58,59^

### Limitations

This study has important limitations. First, given the recency of evidence on the efficacy of SGLT2 inhibitors for CKD, data on the long-term effectiveness of SGLT2 inhibitors are limited. Our base case analysis relied on data from a single randomized clinical trial, DAPA-CKD.^2^ Although other clinical trials have tested the efficacy of SGLT2 inhibitors in CKD, these trial populations have differed in that they have included patients with and without albuminuria (EMPA-KIDNEY)^3^ or included only patients with diabetes (CREDENCE, SCORED),^58,59^ which precluded us from directly incorporating these trial results in our model. To address these limitations, we conducted sensitivity analyses on overall SGLT2 inhibitor effectiveness. Second, our model did not explicitly simulate the prevalence and incidence of key comorbidities, such as diabetes and hypertension, or cardiovascular events, including heart failure hospitalizations. Prior work has studied the cost-effectiveness of CKD screening in these populations,^60,61^ and we did not find differential results according to self-reported diabetes status in our previous analysis.^8^ Cost-effectiveness would likely be more favorable if reductions in nonfatal cardiovascular events (eg, heart failure) were included, yet we are limited by published trial results, which do not report effectiveness on nonfatal cardiovascular outcomes. Third, although we explored more pessimistic assumptions in treatment uptake and adherence in sensitivity analyses, we were unable to consider all clinical implementation barriers for a large population-wide screening program, including heterogeneity in health care access and treatment adherence, which may reduce cost-effectiveness. We leave this as future work. Fourth, although cost-effectiveness thresholds vary by individual, we conducted our analysis using a single cost-effectiveness analysis of $150 000 per QALY gained, which is increasingly used in US cost-effectiveness analyses.^48,62^ Using the long-standing benchmark of $100 000 per QALY gained would alter our assessments on the optimal age of screening initiation. Finally, we did not conduct our analysis from the societal perspective due to data limitations. We expect inclusion of changes to productivity and/or caregiver time would likely yield more favorable cost-effectiveness estimates due to reduced incidence of KRT.^63,64^ Conducting the analysis from the societal perspective is required to fully evaluate societal implications.

## Conclusions

This cost-effectiveness study found that initiation of population-wide screening for CKD followed by treatment with conventional CKD therapy combined with SGLT2 inhibitors at age 55 years was cost-effective for US adults. Initiation at younger ages may have greater population health benefits but incurred additional costs.

## Notes

Supplement 1. eMethods eTable 1. Consolidated Health Economic Evaluation Reporting Standards 2022 checklist eTable 2. Cost-effectiveness results (modal base case) for deterministic sensitivity analysis eTable 3. Probabilistic sensitivity analysis input table eTable 4. Cumulative incidence of KF on KRT (95% UIs) and reduction of cumulative incidence (95% UIs) compared to status quo, by screen-and-treat strategy eTable 5. Projected cases of KF requiring KRT among in U.S. adult population aged 35 to 75y, averted cases compared to status quo, by screen-and-treat strategy eTable 6. Discounted life years and QALYs, expected gains (with 95% UIs) compared to status quo, by screen-and-treat strategy eTable 7. Discounted costs, expected increase in costs (with 95% UIs), by screen-and-treat strategy eTable 8. Discounted costs and QALYs of all considered screen-and-treat strategies sorted by ascending costs eTable 9. Main cost-effectiveness results with discounted costs, QALYs, and ICERs eFigure 1. Univariate sensitivity analysis for q5y screening from 55-75y† (45-year-olds) eFigure 2. Univariate sensitivity analysis for q5y screening from 45-75y† (45-year-olds) eFigure 3. Univariate sensitivity analysis for q5y screening from 55-75y† (55-year-olds) eFigure 4. Univariate sensitivity analysis for q5y screening from 65-75y† (65-year-olds) eFigure 5. Univariate sensitivity analysis for one-time screening at age 75y† (75-year-olds) eTable 10. Cost-effectiveness of selected screen-and-treat strategies under SGLT2 inhibitor price reductions eTable 11. Cost-effectiveness of selected screen-and-treat strategies under SGLT2 inhibitor effectiveness reported in other clinical trials* and SGLT2 inhibitor price reductions eTable 12. Cost-effectiveness of selected screen-and-treat strategies under lower treatment initiation after screening and lower treatment adherence eTable 13. Probabilities of choosing different screen-and-treat strategies across probabilistic sensitivity analysis samples under two willingness-to-pay thresholds eReferences

Supplement 2. Data Sharing Statement
