# Supplementary material for: When to Start Population-Wide Screening for Chronic Kidney Disease: A Cost-Effectiveness Analysis
Source: JAMA Health Forum. 2024 Nov 8;5(11):e243892. doi: 10.1001/jamahealthforum.2024.3892 (PMC11549659; doi:10.1001/jamahealthforum.2024.3892)
Supplement: Supplement 1. — eMethods eTable 1. Consolidated Health Economic Evaluation Reporting Standards 2022 checklist eTable 2. Cost-effectiveness results (modal base case) for deterministic sensitivity analysis eTable 3. Probabilistic sensitivity analysis input table eTable 4. Cumulative incidence of KF on KRT (95% UIs) and reduction of cumulative incidence (95% UIs) compared to status quo, by screen-and-treat strategy eTable 5. Projected cases of KF requiring KRT among in U.S. adult population aged 35 to 75y, averted cases compared to status quo, by screen-and-treat strategy eTable 6. Discounted life years and QALYs, expected gains (with 95% UIs) compared to status quo, by screen-and-treat strategy eTable 7. Discounted costs, expected increase in costs (with 95% UIs), by screen-and-treat strategy eTable 8. Discounted costs and QALYs of all considered screen-and-treat strategies sorted by ascending costs eTable 9. Main cost-effectiveness results with discounted costs, QALYs, and ICERs eFigure 1. Univariate sensitivity analysis for q5y screening from 55-75y† (45-year-olds) eFigure 2. Univariate sensitivity analysis for q5y screening from 45-75y† (45-year-olds) eFigure 3. Univariate sensitivity analysis for q5y screening from 55-75y† (55-year-olds) eFigure 4. Univariate sensitivity analysis for q5y screening from 65-75y† (65-year-olds) eFigure 5. Univariate sensitivity analysis for one-time screening at age 75y† (75-year-olds) eTable 10. Cost-effectiveness of selected screen-and-treat strategies under SGLT2 inhibitor price reductions eTable 11. Cost-effectiveness of selected screen-and-treat strategies under SGLT2 inhibitor effectiveness reported in other clinical trials* and SGLT2 inhibitor price reductions eTable 12. Cost-effectiveness of selected screen-and-treat strategies under lower treatment initiation after screening and lower treatment adherence eTable 13. Probabilities of choosing different screen-and-treat strategies across probabilistic sensitivity analysis samples under two wil [file jamahealthforum-e243892-s001.pdf]

Supplemental Online Content

Cusick MM, Tisdale RL, Chertow GM, et al. When to start population-wide screening for chronic kidney disease: a cost-effectiveness analysis. *JAMA Health Forum*. 2024;5(11):e243892. doi:10.1001/jamahealthforum.2024.3892

eMethods

eTable 1. Consolidated Health Economic Evaluation Reporting Standards 2022 checklist

eTable 2. Cost-effectiveness results (modal base case) for deterministic sensitivity analysis

eTable 3. Probabilistic sensitivity analysis input table

eTable 4. Cumulative incidence of KF on KRT (95% UIs) and reduction of cumulative incidence (95% UIs) compared to status quo, by screen-and-treat strategy

eTable 5. Projected cases of KF requiring KRT among in U.S. adult population aged 35 to 75y, averted cases compared to status quo, by screen-and-treat strategy

eTable 6. Discounted life years and QALYs, expected gains (with 95% UIs) compared to status quo, by screen-and-treat strategy

eTable 7. Discounted costs, expected increase in costs (with 95% UIs), by screen-and-treat strategy

eTable 8. Discounted costs and QALYs of all considered screen-and-treat strategies sorted by ascending costs

eTable 9. Main cost-effectiveness results with discounted costs, QALYs, and ICERs

eFigure 1. Univariate sensitivity analysis for q5y screening from 55-75y† (45-year-olds)

eFigure 2. Univariate sensitivity analysis for q5y screening from 45-75y† (45-year-olds)

eFigure 3. Univariate sensitivity analysis for q5y screening from 55-75y† (55-year-olds)

eFigure 4. Univariate sensitivity analysis for q5y screening from 65-75y† (65-year-olds)

eFigure 5. Univariate sensitivity analysis for one-time screening at age 75y† (75-year-olds)

© 2024 Cusick MM, et al. *JAMA Health Forum*.

eTable 10. Cost-effectiveness of selected screen-and-treat strategies under SGLT2 inhibitor price reductions

eTable 11. Cost-effectiveness of selected screen-and-treat strategies under SGLT2 inhibitor effectiveness reported in other clinical trials* and SGLT2 inhibitor price reductions

eTable 12. Cost-effectiveness of selected screen-and-treat strategies under lower treatment initiation after screening and lower treatment adherence

eTable 13. Probabilities of choosing different screen-and-treat strategies across probabilistic sensitivity analysis samples under two willingness-to-pay thresholds

eReferences

This supplemental material has been provided by the authors to give readers additional information about their work.

eMethods

Calibration procedure and results

We reported details on our calibration procedure and results in the Supplement of Cusick et. al.[1] We estimated age-specific estimates of CKD prevalence, detection, and treatment status using National Health and Examination Survey (NHANES) data. We fit our model to these estimates as calibration targets using Bayesian calibration (sampling importance resampling).

Deterministic sensitivity analysis

We relied on the modal parameter set obtained from calibration to conduct univariate deterministic sensitivity analyses. To confirm the modal parameter set was representative of our main cost-effectiveness results, which were computed using 10,000 probabilistic parameter sets, we computed cost-effectiveness results under the modal calibration and base case mean parameter set. Cost-effectiveness analysis results from the modal parameter set reasonably replicated results our main results (eTable 2).

Probabilistic sensitivity analysis

In probabilistic sensitivity analyses, we examined the probability of preferring different strategies while accounting for all modeled sources of uncertainty, by running the model 10,000 times with different sampled parameter values (eTable 3). eGFR stage-specific quality-of-life weights and costs were correlated with each other to preserve rank-ordering of eGFR stages. SGLT2 inhibitor hazard ratios (HRs) in reducing all-cause mortality and slowing CKD progression were simultaneously sampled from a multivariate lognormal distribution. In deterministic univariate sensitivity analyses, we grouped these parameters and modified them simultaneously.

© 2024 Cusick MM, et al. *JAMA Health Forum.*

eTable 1. Consolidated Health Economic Evaluation Reporting Standards 2022 checklist

	Item	Guidance for Reporting	Reported insection
**TITLE**			
Title	1	Identify the study as an economic evaluation and specify theinterventions being compared.	Page 1-2
**ABSTRACT**			
Abstract	2	Provide a structured summary that highlights context, key methods,results and alternative analyses.	Page 4-5
**INTRODUCTION**			
Background and objectives	3	Give the context for the study, the study question and its practicalrelevance for decision making in policy or practice.	Page 6-8
**METHODS**			
Health economic analysis plan	4	Indicate whether a health economic analysis plan was developed and where available.	NA
Study population	5	Describe characteristics of the study population (such as age range, demographics, socioeconomic, or clinical characteristics).	Page 7
Setting and location	6	Provide relevant contextual information that may influence findings.	Page 8
Comparators	7	Describe the interventions or strategies being compared and why chosen.	Page 7-8
Perspective	8	State the perspective(s) adopted by the study and why chosen.	Page 10
Time horizon	9	State the time horizon for the study and why appropriate.	Page 10
Discount rate	10	Report the discount rate(s) and reason chosen.	Page 10
Selection of outcomes	11	Describe what outcomes were used as the measure(s) of benefit(s)and harm(s).	Page 10-11
Measurement of outcomes	12	Describe how outcomes used to capture benefit(s) and harm(s)were measured.	Page 7-8
Valuation of outcomes	13	Describe the population and methods used to measure and value outcomes.	Page 10-11
Measurement and valuation of resources and costs	14	Describe how costs were valued.	Page 10-11
Currency, price date, and conversion	15	Report the dates of the estimated resource quantities and unit costs, plus thecurrency and year of conversion.	Page 10-11
Rationale and description of model	16	If modelling is used, describe in detail and why used. Report if the model is publicly available and where it can be accessed.	Page 8-10
Analytics and assumptions	17	Describe any methods for analysing or statistically transforming data, anyextrapolation methods, and approaches for validating any model used.	Page 9
Characterizing heterogeneity	18	Describe any methods used for estimating how the results of the studyvary for sub-groups.	NA
Characterizing distributional effects	19	Describe how impacts are distributed across different individuals or adjustments made to reflect priority populations.	NA
Characterizing uncertainty	20	Describe methods to characterize any sources of uncertainty in the analysis.	Page 10-11
Approach to engagement with patients and others affected by the study	21	Describe any approaches to engage patients or service recipients, the general public, communities, or stakeholders (e.g., clinicians or payers) inthe design of the study.	NA
**RESULTS**			
Study parameters	22	Report all analytic inputs (e.g., values, ranges, references) includinguncertainty or distributional assumptions.	Table 1
Summary of main results	23	Report the mean values for the main categories of costs and outcomes ofinterest and summarise them in the most appropriate overall measure.	Page 12-13, Figure 1

© 2024 Cusick MM, et al. *JAMA Health Forum*.

Effect of uncertainty	24	Describe how uncertainty about analytic judgments, inputs, or projections affect findings. Report the effect of choice of discount rate and time horizon, if applicable.	Page 13-15, Figure 1-3
Effect of engagement with patients and others affected by the study	25	Report on any difference patient/service recipient, general public, community, or stakeholder involvement made to the approach or findings of the study	NA
**DISCUSSION**			
Study findings, limitations, generalizability, and current knowledge	26	Report key findings, limitations, ethical or equity considerations not captured, and how these could impact patients, policy, or practice.	Page 15-19
**OTHER RELEVANT INFORMATION**			
Source of funding	27	Describe how the study was funded and any role of the funder in the identification, design, conduct, and reporting of the analysis	Page 20
Conflicts of interest	28	Report authors conflicts of interest according to journal or International Committee of Medical Journal Editors requirements.	Page 20

© 2024 Cusick MM, et al. *JAMA Health Forum.*

eTable 2. Cost-effectiveness results (modal base case) for deterministic sensitivity analysis

Age	Strategy	Costs ($)	QALYs	Incremental Costs ($)	Incremental QALYs	ICER ($/QALY)
35	Status quo*	239,200	19.12			
	Screen 65y*	239,600	19.13	400	0.01	59,300
	Screen q10y 65-75y†	244,900	19.18	5,400	0.05	100,700
	Screen q5y 65-75y†	245,400	19.19	500	0	114,200
	Screen q5y 55-75y†	247,400	19.2	1,900	0.01	136,800
	Screen q5y 45-75y†	249,200	19.21	1,900	0.01	236,500
	Screen q5y 35-75y†	251,000	19.22	1,700	0.01	242,700
45	Status quo*	261,300	16.05			
	Screen 65y*	261,800	16.06	600	0.01	59,200
	Screen q10y 65-75y†	269,100	16.13	7,300	0.07	100,000
	Screen q5y 65-75y†	269,800	16.14	700	0.01	114,100
	Screen q5y 55-75y†	272,400	16.15	2,600	0.02	136,800
	Screen q5y 45-75y†	274,900	16.17	2,500	0.01	228,800
55	Status quo*	266,700	12.7			
	Screen 65y*	267,500	12.71	800	0.01	59,100
	Screen 55y*	267,800	12.71	400	0	92,200
	Screen q10y 65-75y†	277,500	12.81	9,600	0.1	99,700
	Screen q5y 65-75y†	278,400	12.82	1,000	0.01	114,100
	Screen q10y 55-75y†	280,400	12.83	2,000	0.02	131,700
	Screen q5y 55-75y†	282,000	12.84	1,600	0.01	134,900
65	Status quo*	242,900	9.16			
	Screen 65y*	244,000	9.18	1,100	0.02	56500
	Screen q10y 65-75y†	258,300	9.32	14,300	0.14	98600
	Screen q5y 65-75y†	259,700	9.34	1,400	0.01	113900
75	Status quo*	183,900	5.81			
	Screen 75y*	184,900	5.82	1000	0.01	77400
	Screen 75y†	198,900	5.97	14,000	0.15	93700

q10y: every 10 years
q5y: every 5 years
* With conventional CKD therapy comprising of angiotensin-converting enzyme (ACE) inhibitors or angiotensin receptor blocker (ARB) therapy
† With the addition of sodium–glucose cotransporter-2 (SGLT2) inhibitors to conventional CKD therapy

eTable 3. Probabilistic sensitivity analysis input table

Parameters	Value	Range (95% UI)	Distribution
**Screening parameters**			
UACR screening sensitivity [2]	0.87	(0.81, 0.91)	Multivariate normal
UACR screening specificity [2]	0.88	(0.84. 0.91)	Multivariate normal
Cost of UACR screening [3,4]	$52	($40, $66)	Symmetrical beta
Probability of treatment initiation after diagnosis [5,6]	0.75	(0.5,1)	
**Diagnosis parameters**			
Cost of estimated GFR [4,7]	$25	($18, $31)	Symmetrical beta
Cost of retroperitoneal ultrasound [8]	$449	($334, $563)	Symmetrical beta
**Treatment parameters**			
ACE/ARBs – CKD progression reduction – hazards ratio [9–12]	0.81	(0.52, 1)	Lognormal
Monthly cost of ACE/ARBs [4,13]	$36	($27, $46)	Beta
SGLT-2 inhibitors – CKD progression reduction – hazards ratio (persons without diabetes) [14,15]	0.51	(0.34, 0.72)	Multivariate lognormal
SGLT-2 inhibitors – all-cause mortality reduction – hazards ratio (persons without diabetes) [2,35]	0.54	(0.32, 0.86)	Multivariate lognormal
SGLT-2 inhibitors – CKD progression reduction – hazards ratio (persons with diabetes) [14,15]	0.57	(0.45, 0.70)	Multivariate lognormal
SGLT-2 inhibitors – all-cause mortality reduction – hazards ratio (persons with diabetes) [14,15]	0.75	(0.56, 0.98)	Multivariate lognormal
Annual discontinuation rate (SGLT2 inhibitors) [14]	0.051	(0.027, 0.083)	Beta
Monthly cost of SGLT-2 inhibitors [4,13]	$407	($303, $510)	Symmetrical beta
Disutility associated with medication related angioedema adverse event [16]	0.01	(0.0027, 0.02)	Beta
Cost increase from angioedema medication-related adverse event [17]	$4,148	($3,089, $5,209)	Symmetrical beta
Proportion of diagnosed persons who experience an angioedema medication-related serious adverse event [18]	0.1%	(0.01%, 1.0%)	Beta

© 2024 Cusick MM, et al. *JAMA Health Forum*.

Disutility associated with genital infection adverse event [19]	0.001	(0.0002, 0.006)	Beta
Cost increase from genital infection adverse event [20]	$161	($119, $202)	Symmetrical beta
Annual rate of genital infection adverse event [21]	0.037	(0.027, 0.051)	Beta
Disutility associated with euglycemic diabetic ketoacidosis adverse event [22]	0.0098	(0.005, 0.016)	Beta
Cost increase from euglycemic diabetic ketoacidosis adverse event [23]	$32,741	($24,413, $41,115)	Symmetrical beta
Annual rate of euglycemic diabetic ketoacidosis adverse event [24]	0.002	(0.0002, 0.006)	Beta
**Age-specific diabetes prevalence (among those eligible for SGLT2 inhibitor treatment)** [25]			
Diabetes prevalence (30-39-year-olds)	11.4%	(4%, 21.1%)	Binomial
Diabetes prevalence (40-49-year-olds)	28.1%	(17.6%, 38.2%)	Binomial
Diabetes prevalence (50-59-year-olds)	55.5%	(47.9%, 63.0%)	Binomial
Diabetes prevalence (60-69-year-olds)	40.8%	(35.6%, 46.0%)	Binomial
Diabetes prevalence (70-79-year-olds)	43.8%	(39.6%, 47.9%)	Binomial
**CKD mortality parameters**			
Mortality risk - CKD stage G3a - hazard ratio[26]	1.2	(1.1, 1.3)	Symmetrical beta
Mortality risk - CKD stage G3b - hazard ratio[26]	1.8	(1.7, 1.9)	Symmetrical beta
Mortality risk - CKD stage G4 - hazard ratio [26]	3.2	(3.0, 3.4)	Symmetrical beta
Mortality risk - kidney failure not requiring KRT - hazard ratio[26]	3.2	(3.0, 3.4)	Symmetrical beta
Mortality risk - kidney failure not requiring KRT - hazard ratio[26]	5.9	(5.4, 6.4)	Symmetrical beta
**CKD Quality-of-Life adjustments for health states parameters**			
Quality of life adjustment - CKD Stage G2 [15,27]	0.85	(0.7, 0.96)	Beta with induced correlations
Quality of life adjustment - CKD Stage G3a [15,27]	0.81	(0.66, 0.92)	Beta with induced correlations
Quality of life adjustment - CKD Stage G3b [15,27]	0.81	(0.66, 0.92)	Beta with induced correlations
Quality of life adjustment - CKD Stage G4 [15,27]	0.74	(0.61, 0.85)	Beta with induced correlations

© 2024 Cusick MM, et al. *JAMA Health Forum.*

Quality of life adjustment - kidney failure not requiring KRT [15,27]	0.74	(0.61, 0.85)	Beta with induced correlations
Quality of life adjustment – kidney failure requiring KRT [15,27]	0.60	(0.51, 0.68)	Beta with induced correlations
**CKD stage-specific cost parameters**			
Monthly added cost of CKD Stage G3a for overall population [4,28]	$146	($108, $182)	Beta with induced correlations
Monthly added cost of CKD Stage G3b for overall population [4,28]	$394	($293, $492)	Beta with induced correlations
Monthly added cost of CKD Stage G4 for overall population [4,28]	$1,144	($843, $1,441)	Beta with induced correlations
Monthly added cost of kidney failure not requiring KRT for overall population [4,28]	$1,144	($843, $1,441)	Beta with induced correlations
Monthly added cost of kidney failure requiring KRT for overall population [4,28]	$7,512	($5,582, $9,442)	Beta with induced correlations
Monthly added cost of diabetes for overall population (undetected CKD Stage G3a) [4,28]	$110	($82, $139)	Beta with induced correlations
Monthly added cost of diabetes for overall population (undetected CKD Stage G3b) [4,28]	$278	($207, $349)	Beta with induced correlations
Monthly added cost of diabetes for overall population (undetected CKD Stage G4) [4,28]	$643	($479, $810)	Beta with induced correlations
Monthly added cost of diabetes for overall population (undetected kidney failure not requiring KRT) [4,28]	$643	($479, $810)	Beta with induced correlations
Baseline costs[29]	AHRQ* US expenditure table (2013 converted to 2024)	(75%, 125%)	Symmetrical beta
**Calibration parameters**[1,25]			Calibration

© 2024 Cusick MM, et al. *JAMA Health Forum*.

eTable 4. Cumulative incidence of KF on KRT (95% UIs) and reduction of cumulative incidence (95% UIs) compared to status quo, by screen-and-treat strategy

Age	Strategy	Cumulative incidence of KF on KRT	Percentage point reduction in incidence of KF on KRT
35	Status quo*	2.39% [0.67%, 5.13%]	---
	Screen q10y (55-75y)†	1.98% [0.48%, 4.47%]	-0.40 [-1.12, 0.12]
	Screen q5y (55-75y)†	1.94% [0.46%, 4.39%]	-0.45 [-1.20, 0.08]
	Screen q10y (35-75y)†	1.92% [0.46%, 4.35%]	-0.47 [-1.22, 0.06]
	Screen q5y (35-75y)†	1.87% [0.45%, 4.26%]	-0.52 [-1.32, 0.01]
45	Status quo*	2.36% [0.66%, 5.11%]	---
	Screen q10y (55-75y)†	1.96% [0.47%, 4.43%]	-0.40 [-1.12, 0.12]
	Screen q5y (55-75y)†	1.92% [0.46%, 4.35%]	-0.45 [-1.20, 0.08]
	Screen q10y (45-75y)†	1.92% [0.46%, 4.36%]	-0.44 [-1.19, 0.08]
	Screen q5y (45-75y)†	1.88% [0.44%, 4.28%]	-0.49 [-1.26, 0.04]
55	Status quo*	2.33% [0.66%, 5.03%]	---
	Screen q10y (65-75y)†	2.02% [0.49%, 4.49%]	-0.31 [-0.97, 0.23]
	Screen q5y (65-75y)†	1.99% [0.48%, 4.43%]	-0.34 [-1.03, 0.20]
	Screen q10y (55-75y)†	1.92% [0.46%, 4.33%]	-0.41 [-1.14, 0.12]
	Screen q5y (55-75y)†	1.87% [0.45%, 4.23%]	-0.45 [-1.22, 0.09]
65	Status quo*	2.30% [0.63%, 4.97%]	---
	Screen 75y†	2.15% [0.57%, 4.68%]	-0.14 [-0.69, 0.40]
	Screen q10y (65-75y)†	1.97% [0.51%, 4.39%]	-0.32 [-1.01, 0.25]
	Screen q5y (65-75y)†	1.94% [0.49%, 4.33%]	-0.35 [-1.07, 0.21]
75	Status quo*	2.00% [0.62%, 4.31%]	---
	Screen 75y†	1.89% [0.54%, 4.14%]	-0.11 [-0.11, 0.45]

q10y: every 10 years
q5y: every 5 years
* With conventional CKD therapy comprising of angiotensin-converting enzyme (ACE) inhibitors or angiotensin receptor blocker (ARB) therapy
† With the addition of sodium–glucose cotransporter-2 (SGLT2) inhibitors to conventional CKD therapy

© 2024 Cusick MM, et al. *JAMA Health Forum.*

eTable 5. Projected cases of KF requiring KRT among in U.S. adult population aged 35 to 75y, averted cases compared to status quo, by screen-and-treat strategy

Strategy	Cases of KF requiring KRT	Averted cases	% reduction
Status quo*	3,662,000	---	---
Screen q10y (55-75y)†	3,091,000	571,000	16%
Screen q5y (55-75y)†	3,054,000	608,000	17%
Screen q10y (35-75y)†	3,060,000	602,000	16%
Screen q5y (35-75y)†	3,000,000	662,000	18%

q10y: every 10 years
q5y: every 5 years
* With conventional CKD therapy comprising of angiotensin-converting enzyme (ACE) inhibitors or angiotensin receptor blocker (ARB) therapy
† With the addition of sodium–glucose cotransporter-2 (SGLT2) inhibitors to conventional CKD therapy

© 2024 Cusick MM, et al. *JAMA Health Forum.*

eTable 6. Discounted life years and QALYs, expected gains (with 95% UIs) compared to status quo, by screen-and-treat strategy

Age	Strategy	LYs	LY Gain	QALYs	QALY Gain
35	Status quo*	23.81 [23.70, 23.90]	---	19.12 [17.75, 20.09]	---
	Screen q10y (55-75y)†	23.93 [23.81, 24.04]	0.12 [0.05, 0.19]	19.20 [17.81, 20.17]	0.08 [0.04, 0.13]
	Screen q5y (55-75y)†	23.94 [23.82, 24.05]	0.13 [0.05, 0.20]	19.21 [17.81, 20.18]	0.09 [0.04, 0.14]
	Screen q10y (35-75y)†	23.94 [23.82, 24.05]	0.13 [0.06, 0.21]	19.22 [17.83, 20.19]	0.09 [0.04, 0.15]
	Screen q5y (35-75y)†	23.95 [23.83, 24.07]	0.14 [0.06, 0.22]	19.22 [17.83, 20.20]	0.10 [0.05, 0.16]
45	Status quo*	20.87 [20.72, 20.99]	---	16.03 [14.54, 17.08]	---
	Screen q10y (55-75y)†	21.03 [20.87, 21.18]	0.16 [0.07, 0.26]	16.14 [14.62, 17.2]	0.11 [0.05, 0.18]
	Screen q5y (55-75y)†	21.04 [20.88, 21.19]	0.17 [0.07, 0.28]	16.14 [14.63, 17.21]	0.12 [0.05, 0.19]
	Screen q10y (45-75y)†	21.04 [20.88, 21.19]	0.17 [0.07, 0.28]	16.15 [14.63, 17.21]	0.12 [0.06, 0.19]
	Screen q5y (45-75y)†	21.05 [20.89, 21.21]	0.18 [0.08, 0.30]	16.16 [14.64, 17.22]	0.13 [0.06, 0.21]
55	Status quo*	17.29 [17.09, 17.45]	---	12.62 [11.1, 13.7]	---
	Screen q10y (65-75y)†	17.47 [17.26, 17.66]	0.18 [0.08, 0.30]	12.74 [11.21, 13.83]	0.12 [0.05, 0.20]
	Screen q5y (65-75y)†	17.48 [17.27, 17.68]	0.20 [0.07, 0.32]	12.75 [11.22, 13.84]	0.13 [0.06, 0.22]
	Screen q10y (55-75y)†	17.51 [17.29, 17.71]	0.22 [0.09, 0.36]	12.77 [11.23, 13.86]	0.15 [0.07, 0.25]
	Screen q5y (55-75y)†	17.52 [17.30, 17.73]	0.24 [0.10, 0.38]	12.78 [11.24, 13.88]	0.16 [0.08, 0.27]
65	Status quo*	13.20 [12.93, 13.43]	---	9.05 [7.66, 10.07]	---
	Screen 75y†	13.38 [13.09, 13.62]	0.17 [0.06, 0.29]	9.16 [7.75, 10.19]	0.11 [0.04, 0.19]
	Screen q10y (65-75y)†	13.47 [13.17, 13.74]	0.27 [0.11, 0.43]	9.23 [7.8, 10.27]	0.18 [0.08, 0.29]
	Screen q5y (65-75y)†	13.49 [13.19, 13.76]	0.28 [0.12, 0.47]	9.24 [7.81, 10.28]	0.19 [0.08, 0.31]
75	Status quo*	8.70 [8.34, 9.00]	---	5.66 [4.67, 6.42]	---
	Screen 75y†	8.97 [8.58, 9.29]	0.26 [0.10, 0.44]	5.83 [4.8, 6.61]	0.17 [0.06, 0.28]

q10y: every 10 years
q5y: every 5 years
* With conventional CKD therapy comprising of angiotensin-converting enzyme (ACE) inhibitors or angiotensin receptor blocker (ARB) therapy
† With the addition of sodium–glucose cotransporter-2 (SGLT2) inhibitors to conventional CKD therapy

© 2024 Cusick MM, et al. *JAMA Health Forum*.

eTable 7. Discounted costs, expected increase in costs (with 95% UIs), by screen-and-treat strategy

Age	Strategy	Healthcare sector costs ($)	Increase in costs ($)
35	Status quo*	241,100 [182,200, 301,800]	---
	Screen q10y (55-75y)†	248,500 [189,000, 310,000]	7,400 [4700, 10,500]
	Screen q5y (55-75y)†	249,300 [189,800, 310,700]	8,300 [5.300, 11600]
	Screen q10y (35-75y)†	251,300 [191,700, 312,900]	10,300 [6,800, 14,200]
	Screen q5y (35-75y)†	253,000 [193,300, 314,700]	11,900 [8,000, 16,200]
45	Status quo*	265,600 [201,500, 331,800]	---
	Screen q10y (55-75y)†	273,800 [208,800, 340,800]	10,100 [6,400, 14,400]
	Screen q5y (55-75y)†	274,900 [209,800, 341,900]	11,200 [7,300, 15,800]
	Screen q10y (45-75y)†	275,800 [210,600, 342,800]	12,100 [7,800, 16,900]
	Screen q5y (45-75y)†	277,400 [212,200, 344,500]	13,700 [9,100, 19,000]
55	Status quo*	269,400 [204,600, 336,200]	---
	Screen q10y (65-75y)†	280,200 [214,300, 348,100]	10,800 [6,700, 15,600]
	Screen q5y (65-75y)†	281,100 [215,200, 349,200]	11,700 [7,300, 16,900]
	Screen q10y (55-75y)†	283,300 [216,900, 351,500]	13,900 [8,900, 19,800]
	Screen q5y (55-75y)†	284,900 [218,400, 353,000]	15,500 [10,00, 21,800]
65	Status quo*	245,300 [187,300, 305,000]	---
	Screen 75y†	255,100 [195,900, 315,900]	9,800 [5,700, 14,600]
	Screen q10y (65-75y)†	26,0800 [201,000, 322,200]	16,900 [10,500, 24,300]
	Screen q5y (65-75y)†	262,200 [202,200, 323,800]	16,900 [10,500, 24,300]
75	Status quo*	185,100 [142,400, 229,400]	---
	Screen 75y†	199,800 [155,000, 245,600]	14,700 [8,600, 21,900]

q10y: every 10 years
q5y: every 5 years
* With conventional CKD therapy comprising of angiotensin-converting enzyme (ACE) inhibitors or angiotensin receptor blocker (ARB) therapy
† With the addition of sodium–glucose cotransporter-2 (SGLT2) inhibitors to conventional CKD therapy

© 2024 Cusick MM, et al. *JAMA Health Forum.*

eTable 8. Discounted costs and QALYs of all considered screen-and-treat strategies sorted by ascending costs

Age	Strategy	QALYs	Costs ($)
35	Status quo*	19.12 [17.75, 20.09]	241,100 [182,200, 301,800]
	Screen 75y*	19.12 [17.75, 20.09]	241,300 [182,500, 302,200]
	Screen 65y*	19.13 [17.76, 20.09]	241,400 [182,600, 302,400]
	Screen q10y 65-75y*	19.13 [17.76, 20.09]	241,600 [182,700, 302,500]
	Screen 55y*	19.13 [17.76, 20.09]	241,600 [182,700, 302,600]
	Screen q5y 65-75y*	19.13 [17.76, 20.09]	241,700 [182,800, 302,700]
	Screen 45y*	19.13 [17.76, 20.09]	241,900 [183,000, 302,900]
	Screen q10y 55-75y*	19.13 [17.76, 20.1]	242,000 [183,100, 303,000]
	Screen 35y*	19.13 [17.76, 20.09]	242,200 [183,400, 303,200]
	Screen q5y 55-75y*	19.14 [17.76, 20.1]	242,300 [183,500, 303,300]
	Status quo†	19.14 [17.76, 20.1]	242,500 [183,700, 303,500]
	Screen q10y 45-75y*	19.14 [17.77, 20.1]	242,600 [183,800, 303,600]
	Screen q5y 45-75y*	19.14 [17.76, 20.1]	243,200 [184,400, 304,200]
	Screen q10y 35-75y*	19.14 [17.77, 20.1]	243,400 [184,700, 304,400]
	Screen q5y 35-75y*	19.14 [17.77, 20.1]	244,400 [185,800, 305,500]
	Screen 75y†	19.16 [17.78, 20.13]	244,700 [185,700, 305,900]
	Screen 65y†	19.18 [17.79, 20.15]	245,900 [186,800, 307,300]
	Screen 35y†	19.17 [17.79, 20.14]	246,600 [187,500, 307,800]
	Screen 55y†	19.18 [17.79, 20.15]	246,600 [187,300, 308,000]
	Screen q10y 65-75y†	19.19 [17.8, 20.16]	246,800 [187,500, 308,300]
	Screen 45y†	19.18 [17.79, 20.15]	246,800 [187,600, 308,200]
	Screen q5y 65-75y†	19.19 [17.8, 20.16]	247,300 [188,000, 308,700]
	Screen q10y 55-75y†	19.20 [17.81, 20.17]	248,500 [189,000, 310,000]
	Screen q5y 55-75y†	19.21 [17.81, 20.18]	249,300 [189,800, 310,700]
	Screen q10y 45-75y†	19.21 [17.82, 20.18]	250,000 [190,300, 311,500]
	Screen q5y 45-75y†	19.22 [17.82, 20.19]	251,200 [191,500, 312,800]
	Screen q10y 35-75y†	19.22 [17.83, 20.19]	251,300 [191,700, 312,900]
	Screen q5y 35-75y†	19.22 [17.83, 20.2]	253,000 [193,300, 314,700]
45	Status quo*	16.03 [14.54, 17.08]	263,700 [199,800, 329,800]
	Screen 75y*	16.03 [14.54, 17.08]	264,000 [200,000, 330,100]
	Screen 65y*	16.03 [14.54, 17.09]	264,200 [200,300, 330,400]
	Screen q10y 65-75y*	16.04 [14.54, 17.09]	264,400 [200,400, 330,600]
	Screen 55y*	16.04 [14.55, 17.09]	264,500 [200,500, 330,700]
	Screen q5y 65-75y*	16.04 [14.54, 17.09]	264,500 [200,500, 330,800]
	Screen 45y*	16.04 [14.55, 17.09]	264,800 [200,800, 331,100]
	Screen q10y 55-75y*	16.04 [14.55, 17.09]	264,900 [200,900, 331,200]

© 2024 Cusick MM, et al. *JAMA Health Forum*.

	Screen q5y 55-75y*	16.04 [14.55, 17.1]	265,400 [201,500, 331,700]
	Status quo†	16.05 [14.55, 17.1]	265,600 [201,500, 331,800]
	Screen q10y 45-75y*	16.05 [14.56, 17.1]	265,700 [201,900, 332,100]
	Screen q5y 45-75y*	16.05 [14.56, 17.1]	266,500 [202,600, 332,900]
	Screen 75y†	16.08 [14.58, 17.14]	268,600 [204,400, 335,200]
	Screen 65y†	16.10 [14.6, 17.16]	270,300 [205,700, 337,000]
	Screen 55y†	16.11 [14.6, 17.17]	271,200 [206,300, 337,900]
	Screen 45y†	16.10 [14.6, 17.16]	271,500 [206,700, 338,100]
	Screen q10y 65-75y†	16.11 [14.6, 17.17]	271,500 [206,700, 338,300]
	Screen q5y 65-75y†	16.12 [14.61, 17.18]	272,200 [207,300, 339,000]
	Screen q10y 55-75y†	16.14 [14.62, 17.2]	273,800 [208,800, 340,800]
	Screen q5y 55-75y†	16.14 [14.63, 17.21]	274,900 [209,800, 341,900]
	Screen q10y 45-75y†	16.15 [14.63, 17.21]	275,800 [210,600, 342,800]
	Screen q5y 45-75y†	16.16 [14.64, 17.22]	277,400 [212,200, 344,500]
55	Status quo*	12.62 [11.1, 13.7]	269,400 [204,600, 336,200]
	Screen 75y*	12.62 [11.1, 13.7]	269,800 [204,900, 336,700]
	Screen 65y*	12.63 [11.11, 13.71]	270,100 [205,200, 337,000]
	Screen q10y 65-75y*	12.63 [11.11, 13.71]	270,300 [205,400, 337,300]
	Screen 55y*	12.63 [11.12, 13.71]	270,400 [205,500, 337,300]
	Screen q5y 65-75y*	12.63 [11.11, 13.71]	270,600 [205,700, 337,500]
	Screen q10y 55-75y*	12.64 [11.12, 13.72]	271,000 [206,200, 338,200]
	Screen q5y 55-75y*	12.64 [11.12, 13.72]	271,600 [206,900, 338,900]
	Status quo†	12.65 [11.12, 13.72]	271,900 [206,900, 339,100]
	Screen 75y†	12.69 [11.16, 13.78]	276,200 [211,000, 343,600]
	Screen 65y†	12.72 [11.19, 13.81]	278,500 [212,800, 346,100]
	Screen 55y†	12.73 [11.2, 13.82]	279,600 [213,700, 347,400]
	Screen q10y 65-75y†	12.74 [11.21, 13.83]	280,200 [214,300, 348,100]
	Screen q5y 65-75y†	12.75 [11.22, 13.84]	281,100 [215,200, 349,200]
	Screen q10y 55-75y†	12.77 [11.23, 13.86]	283,300 [216,900, 351,500]
	Screen q5y 55-75y†	12.78 [11.24, 13.88]	284,900 [218,400, 353,000]
65	Status quo*	9.05 [7.66, 10.07]	245,300 [187,300, 305,000]
	Screen 75y*	9.06 [7.66, 10.07]	245,900 [187,900, 305,700]
	Screen 65y*	9.07 [7.67, 10.08]	246,300 [188,300, 306,200]
	Screen q10y 65-75y*	9.07 [7.67, 10.09]	246,700 [188,700, 306,600]
	Screen q5y 65-75y*	9.08 [7.68, 10.09]	247,000 [189,100, 307,100]
	Status quo†	9.09 [7.69, 10.11]	248,800 [190,600, 308,900]
	Screen 75y†	9.16 [7.75, 10.19]	255,100 [195,900, 315,900]
	Screen 65y†	9.20 [7.78, 10.23]	258,400 [198,700, 319,500]
	Screen q10y 65-75y†	9.23 [7.8, 10.27]	260,800 [201,000, 322,200]
	Screen q5y 65-75y†	9.24 [7.81, 10.28]	262,200 [202,200, 323,800]

© 2024 Cusick MM, et al. *JAMA Health Forum*.

75	Status quo*	5.66 [4.67, 6.42]	185,100 [142,400, 229,400]
	Screen 75y*	5.67 [4.68, 6.43]	186,100 [143,300, 230,500]
	Status quo†	5.70 [4.71, 6.46]	189,600 [146,500, 234,300]
	Screen 75y†	5.83 [4.8, 6.61]	199,800 [155,000, 245,600]

q10y: every 10 years
q5y: every 5 years
* With conventional CKD therapy comprising of angiotensin-converting enzyme (ACE) inhibitors or angiotensin receptor blocker (ARB) therapy
† With the addition of sodium–glucose cotransporter-2 (SGLT2) inhibitors to conventional CKD therapy

© 2024 Cusick MM, et al. *JAMA Health Forum*.

eTable 9. Main cost-effectiveness results with discounted costs, QALYs, and ICERs

Age	Strategy	Costs ($)	QALYs	Incremental Costs ($)	Incremental QALYs	ICER ($/QALY)
35	Status quo*	241,100	19.12			
	Screen 65y*	241,400	19.13	400	0.01	59,400
	Screen 55y*	241,600	19.13	200	0	76,100
	Screen 65y†	245,900	19.18	4,300	0.05	91,400
	Screen q10y 65-75y†	246,800	19.19	900	0.01	92,500
	Screen q5y 65-75y†	247,300	19.19	500	0	107,300
	Screen q10y 55-75y†	248,500	19.20	1,200	0.01	111,900
	Screen q5y 55-75y†	249,300	19.21	800	0.01	128,400
	Screen q5y 45-75y†	251,200	19.22	1,900	0.01	226,700
	Screen q5y 35-75y†	253,000	19.22	1,800	0.01	268,200
45	Status quo*	263,700	16.03			
	Screen 65y*	264,200	16.03	500	0.01	59,300
	Screen 55y*	264,500	16.04	300	0	76,000
	Screen 65y†	270,300	16.10	5,800	0.06	91,000
	Screen q10y 65-75y†	271,500	16.11	1,200	0.01	92,500
	Screen q5y 65-75y†	272,200	16.12	700	0.01	107,200
	Screen q10y 55-75y†	273,800	16.14	1,600	0.01	111,800
	Screen q5y 55-75y†	274,900	16.14	1,100	0.01	128,300
	Screen q5y 45-75y†	277,400	16.16	2,500	0.01	219,400
55	Status quo*	269,400	12.62			
	Screen 65y*	270,100	12.63	700	0.01	59,200
	Screen 55y*	270,400	12.63	300	0	67,300
	Screen q10y 65-75y†	278,500	12.72	8,100	0.09	91,700
	Screen q5y 65-75y†	280,200	12.74	1,600	0.02	92,500
	Screen q10y 55-75y†	283,300	12.77	3,100	0.03	107,100
	Screen q5y 55-75y†	284,900	12.78	1,600	0.01	128,300
65	Status quo*	245,300	9.05			
	Screen 65y*	246,300	9.07	1,000	0.02	56,300
	Screen 65y†	258,400	9.20	12,100	0.13	90,500
	Screen q10y 65-75y†	260,800	9.23	2,400	0.03	92,300
	Screen q5y 65-75y†	262,200	9.24	1,400	0.01	107,100
75	Status quo*	185,100	5.66			
	Screen 75y*	186,100	5.67	1,000	0.01	76,500
	Screen 75y†	199,800	5.83	13,700	0.15	89,300

q10y: every 10 years
q5y: every 5 years

© 2024 Cusick MM, et al. *JAMA Health Forum*.

[*] With conventional CKD therapy comprising of angiotensin-converting enzyme (ACE) inhibitors or angiotensin receptor blocker (ARB) therapy
[†] With the addition of sodium–glucose cotransporter-2 (SGLT2) inhibitors to conventional CKD therapy

© 2024 Cusick MM, et al. *JAMA Health Forum*.

eFigure 1. Univariate sensitivity analysis for q5y screening from 55-75y† (45-year-olds)

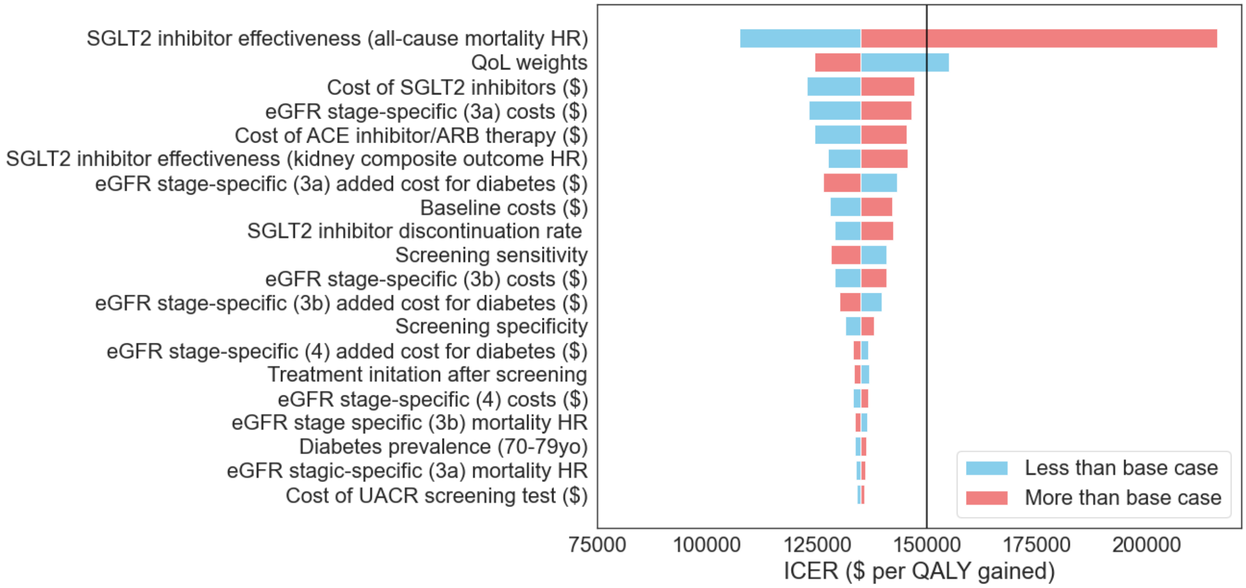

q5y: every 5 years
q10y: every 10 years
† With the addition of sodium–glucose cotransporter-2 (SGLT2) inhibitors to conventional CKD therapy (angiotensin-converting enzyme [ACE] inhibitors or angiotensin receptor blocker [ARB] therapy)
Comparison strategy: q10y screening 55-75y†

© 2024 Cusick MM, et al. *JAMA Health Forum*.

eFigure 2. Univariate sensitivity analysis for q5y screening from 45-75y† (45-year-olds)

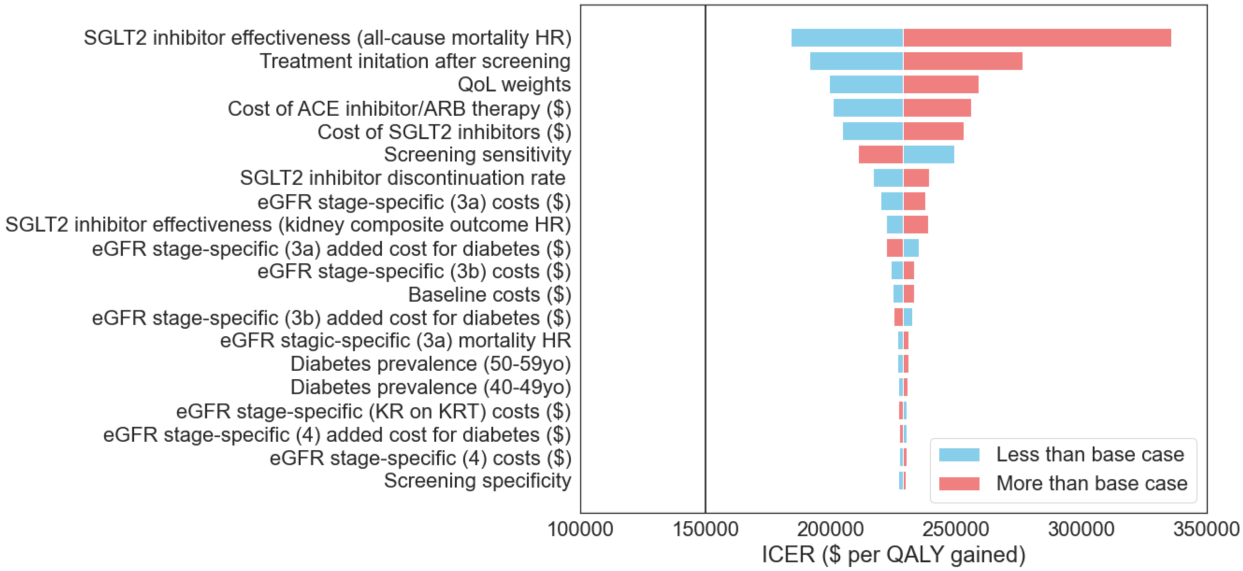

q5y: every 5 years
† With the addition of sodium–glucose cotransporter-2 (SGLT2) inhibitors to conventional CKD therapy (angiotensin-converting enzyme [ACE] inhibitors or angiotensin receptor blocker [ARB] therapy)
Comparison strategy: q5y screening 55-75y†

© 2024 Cusick MM, et al. *JAMA Health Forum*.

eFigure 3. Univariate sensitivity analysis for q5y screening from 55-75y† (55-year-olds)

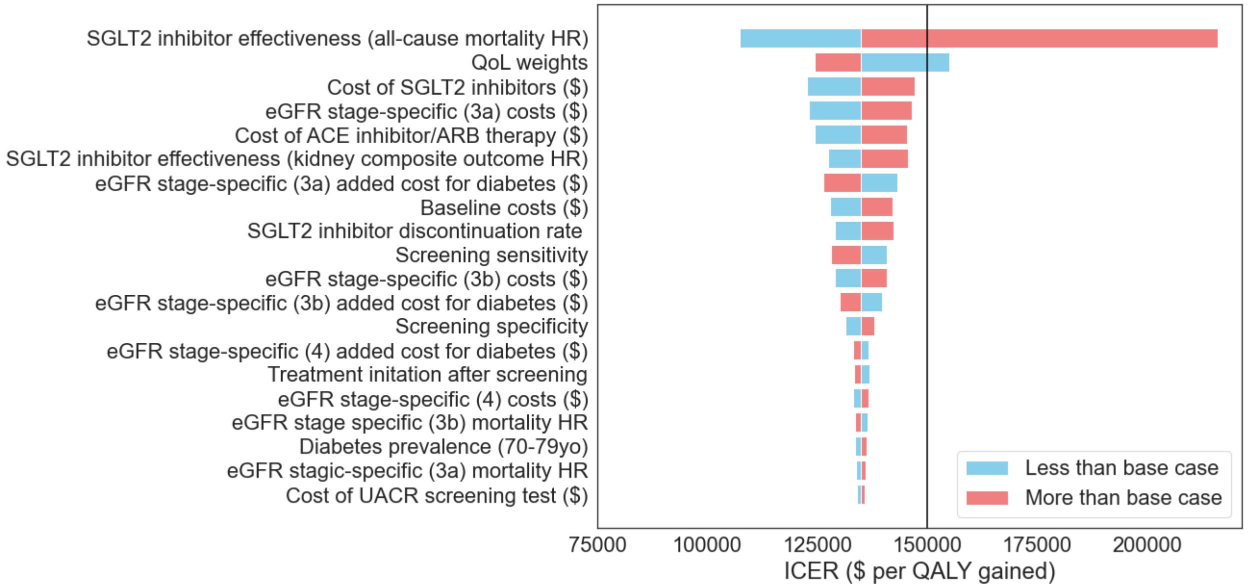

q5y: every 5 years
q10y: every 10 years
† With the addition of sodium–glucose cotransporter-2 (SGLT2) inhibitors to conventional CKD therapy (angiotensin-converting enzyme [ACE] inhibitors or angiotensin receptor blocker [ARB] therapy)
Comparison strategy: q10y screening 55-75y†

© 2024 Cusick MM, et al. *JAMA Health Forum*.

eFigure 4. Univariate sensitivity analysis for q5y screening from 65-75y† (65-year-olds)

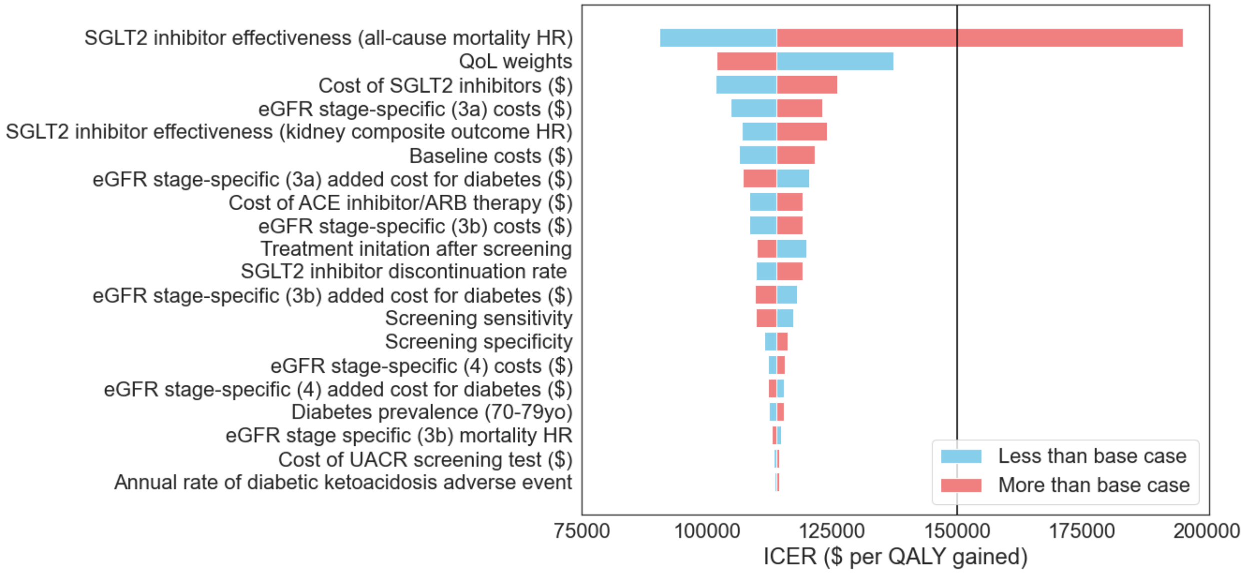

q5y: every 5 years

† With the addition of sodium–glucose cotransporter-2 (SGLT2) inhibitors to conventional CKD therapy (angiotensin-converting enzyme [ACE] inhibitors or angiotensin receptor blocker [ARB] therapy)

Comparison strategy: q10y screening 65-75y†

eFigure 5. Univariate sensitivity analysis for one-time screening from at age 75† (75-year-olds)

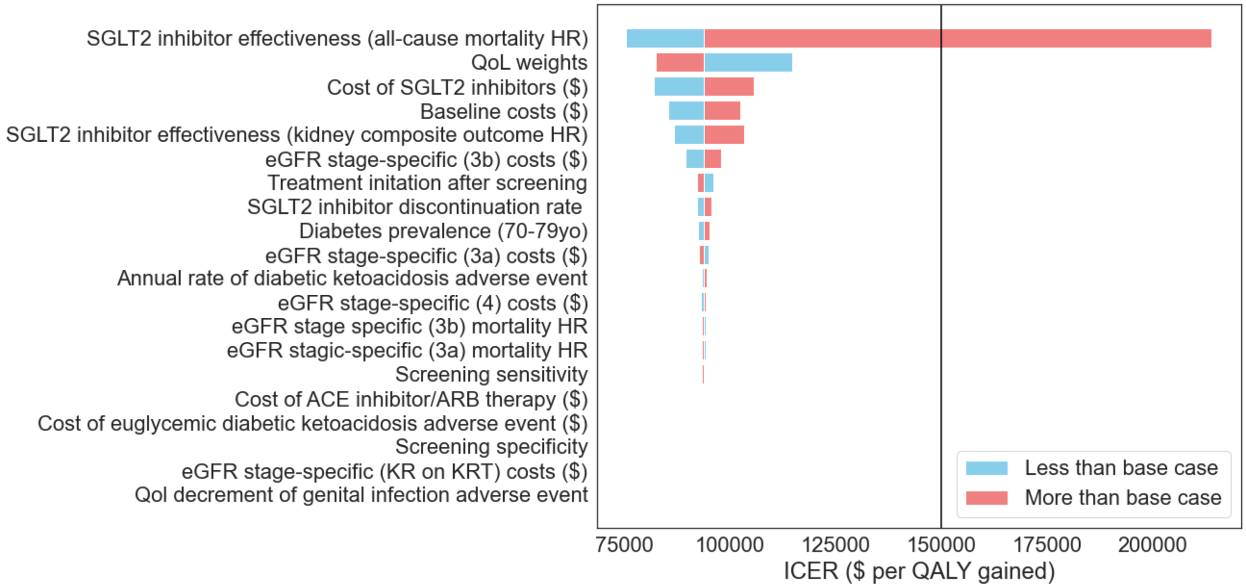

q5y: every 5 years

† With the addition of sodium–glucose cotransporter-2 (SGLT2) inhibitors to conventional CKD therapy (angiotensin-converting enzyme [ACE] inhibitors or angiotensin receptor blocker [ARB] therapy)

Comparison strategy: Status quo with conventional CKD therapy

© 2024 Cusick MM, et al. *JAMA Health Forum*.

eTable 10. Cost-effectiveness of selected screen-and-treat strategies under SGLT2 inhibitor price reductions

		ICERs ($/QALY) under different price reductions			
Age	Strategy	0% ($407)	25% ($305)	50% ($204)	75% ($102)
35	Screen q5y (55-75y)†	$135,000	$123,300	$111,600	$99,900
	Screen q5y (35-75y)†	$242,700	$229,700	$216,800	$203,800
45	Screen q5y (55-75y)†	$134,900	$123,300	$111,600	$99,800
	Screen q5y (45-75y)†	$228,800	$205,700	$182,600	$159,400
55	Screen q10y (55-75y)†	$131,700	$111,000	$90,400	$69,300
	Screen q5y (55-75y)†	$134,800	$123,200	$111,500	$99,800
65	Screen q5y (65-75y)†	$114,000	$102,300	$90,700	$79,200
75	Screen 75y†	$93,700	$82,400	$71,100	$59,700

q10y: every 10 years
q5y: every 5 years
† With the addition of sodium–glucose cotransporter-2 (SGLT2) inhibitors to conventional CKD therapy (angiotensin-converting enzyme [ACE] inhibitors or angiotensin receptor blocker [ARB] therapy)

eTable 11. Cost-effectiveness of selected screen-and-treat strategies under SGLT2 inhibitor effectiveness reported in other clinical trials* and SGLT2 inhibitor price reductions

		ICERs ($/QALY) under different effectiveness reductions			
Age	Strategy	0% ($407)	25% ($305)	50% ($204)	75% ($102)
35	Screen q5y (55-75y)†	$208,400	$189,200	$169,900	$150,700
	Screen q5y (35-75y)†	$284,100	$268,000	$253,000	$238,000
45	Screen q5y (55-75y)†	$208,400	$189,200	$169,900	$150,700
	Screen q5y (45-75y)†	$319,100	$286,300	$253,500	$220,800
55	Screen q10y (55-75y)†	$131,700	$111,000	$90,400	$69,300
	Screen q5y (55-75y)†	$208,300	$189,100	$169,800	$150,700
65	Screen q5y (65-75y)†	$186,100	$165,100	$144,000	$122,900
75	Screen 75y†	$187,500	$157,800	$128,200	$98,700

q10y: every 10 years
q5y: every 5 years
*all-cause mortality: (non-diabetic HR: 0.84, diabetic HR: 0.87), kidney composite outcome: (non-diabetic HR: 0.69, diabetic HR: 0.62)
† With the addition of sodium–glucose cotransporter-2 (SGLT2) inhibitors to conventional CKD therapy (angiotensin-converting enzyme [ACE] inhibitors or angiotensin receptor blocker [ARB] therapy)

© 2024 Cusick MM, et al. *JAMA Health Forum.*

eTable 12. Cost-effectiveness of selected screen-and-treat strategies under lower treatment initiation after screening and lower treatment adherence

		ICERs ($/QALY)		
Age	Intervention	0% change<br>initiation: 0.75<br>annual discontinuation: 5%	15% change<br>initiation: 0.54<br>annual discontinuation:7%	25% change<br>initiation: 0.33<br>annual discontinuation: 10%
35	Screen q5y (55-75y)†	$135,000	$140,600	$150,800
	Screen q5y (35-75y)†	$242,700	$231,500	$228,400
45	Screen q5y (55-75y)†	$134,900	$140,500	$150,800
	Screen q5y (45-75y)†	$228,800	$206,500	$192,100
55	Screen q10y (55-75y)†	$131,700	$120,200	$110,300
	Screen q5y (55-75y)†	$134,800	$140,400	$150,700
65	Screen q5y (65-75y)†	$114,000	$121,200	$134,600
75	Screen 75y†	$93,700	$96,400	$101,200

q10y: every 10 years
q5y: every 5 years
† With the addition of sodium–glucose cotransporter-2 (SGLT2) inhibitors to conventional CKD therapy (angiotensin-converting enzyme [ACE] inhibitors or angiotensin receptor blocker [ARB] therapy)

© 2024 Cusick MM, et al. *JAMA Health Forum.*

eTable 13. Probabilities of choosing different screen-and-treat strategies across probabilistic sensitivity analysis samples under two willingness-to-pay thresholds

Age	Strategy	$100,000/QALY	$150,000/QALY
35	Status quo*	9.77%	0.21%
	Status quo†	0.32%	0.52%
	Start at 35y†	0.32%	7.98%
	Start at 45y†	0.12%	2.68%
	Start at 55y†	18.65%	63.24%
	Start at 65y†	34.51%	15.56%
	Start at 75y†	3.70%	0.42%
	Other*	32.93%	9.39%
45	Status quo*	4.55%	0.18%
	Status quo†	5.11%	0.55%
	Start at 45y†	0.49%	10.98%
	Start at 55y†	18.32%	63.76%
	Start at 65y†	34.84%	15.56%
	Start at 75y†	3.79%	0.45%
	Other*	32.40%	8.52%
55	Status quo*	4.37%	0.17%
	Status quo†	4.95%	0.55%
	Start at 55y†	22.93%	78.23%
	Start at 65y†	31.93%	13.19%
	Start at 75y†	3.90%	0.45%
	Other*	31.93%	7.41%
65	Status quo*	4.84%	0.20%
	Status quo†	4.02%	0.49%
	Start at 65y†	59.42%	93.79%
	Start at 75y†	2.67%	0.27%
	Other*	29.05%	5.25%
75	Status quo*	9.83%	0.39%
	Status quo†	2.31%	0.53%
	Start at 75y†	65.27%	95.31%
	Other*	22.59%	3.77%

* With conventional CKD therapy comprising of angiotensin-converting enzyme (ACE) inhibitors or angiotensin receptor blocker (ARB) therapy
† With the addition of sodium–glucose cotransporter-2 (SGLT2) inhibitors to conventional CKD therapy
Start at 35y: refers to any screen-and-treat strategy that is initiated at age 35 regardless of the frequency of screening (one-time, q10y, q5y) with the addition of SGLT2 inhibitors to conventional CKD therapy
Other: refers to any screen-and-treat strategy initiated at any age and frequency with the use of conventional CKD therapy alone

© 2024 Cusick MM, et al. *JAMA Health Forum*.

© 2024 Cusick MM, et al. *JAMA Health Forum*.

eReferences

1. Cusick MM, Tisdale RL, Chertow GM, Owens DK, Goldhaber-Fiebert JD. Population-Wide Screening for Chronic Kidney Disease. *Ann Intern Med*. 2023;176(6):788-797. doi:10.7326/M22-3228

2. Wu HY, Peng YS, Chiang CK, et al. Diagnostic Performance of Random Urine Samples Using Albumin Concentration vs Ratio of Albumin to Creatinine for Microalbuminuria Screening in Patients With Diabetes Mellitus: A Systematic Review and Meta-analysis. *JAMA Internal Medicine*. 2014;174(7):1108-1115. doi:10.1001/jamainternmed.2014.1363

3. Creatinine, 24-Hour Urine. Find Lab Tests Online. Accessed June 27, 2022. https://www.findlabtest.com/lab-test/kidney-function-test/creatinine-24-hour-urine-quest-381

4. Sanders GD, Neumann PJ, Basu A, et al. Recommendations for Conduct, Methodological Practices, and Reporting of Cost-effectiveness Analyses: Second Panel on Cost-Effectiveness in Health and Medicine. *JAMA*. 2016;316(10):1093. doi:10.1001/jama.2016.12195

5. Golan L, Birkmeyer JD, Welch HG. The Cost-Effectiveness of Treating All Patients with Type 2 Diabetes with Angiotensin-Converting Enzyme Inhibitors. *Ann Intern Med*. 1999;131(9):660-667. doi:10.7326/0003-4819-131-9-199911020-00005

6. Boulware LE, Jaar BG, Tarver-Carr ME, Brancati FL, Powe NR. Screening for Proteinuria in US AdultsA Cost-effectiveness Analysis. *JAMA*. 2003;290(23):3101-3114. doi:10.1001/jama.290.23.3101

7. Serum Creatinine Test Cost. Find Lab Tests Online. Accessed June 27, 2022. https://www.findlabtest.com/lab-test/kidney-function-test/serum-creatinine-test-cost-quest-375

8. Abdominal Ultrasound Cost and Procedure Comparison | NCH. NewChoiceHealth.com. Accessed June 28, 2022. https://www.newchoicehealth.com/procedures/abdominal-ultrasound

9. Ruggenenti P, Perna A, Gherardi G, et al. Renoprotective properties of ACE-inhibition in non-diabetic nephropathies with non-nephrotic proteinuria. *The Lancet*. 1999;354(9176):359-364. doi:10.1016/S0140-6736(98)10363-X

10. Brenner BM, Cooper ME, de Zeeuw D, et al. Effects of Losartan on Renal and Cardiovascular Outcomes in Patients with Type 2 Diabetes and Nephropathy. *N Engl J Med*. 2001;345(12):861-869. doi:10.1056/NEJMoa011161

11. Hou FF, Zhang X, Zhang GH, et al. Efficacy and Safety of Benazepril for Advanced Chronic Renal Insufficiency. *New England Journal of Medicine*. 2006;354(2):131-140. doi:10.1056/NEJMoa053107

12. Hou FF, Xie D, Zhang X, et al. Renoprotection of Optimal Antiproteinuric Doses (ROAD) Study: A Randomized Controlled Study of Benazepril and Losartan in Chronic Renal Insufficiency. *JASN*. 2007;18(6):1889-1898. doi:10.1681/ASN.2006121372

13. US Department of Veterans Affairs. VA Federal Supply Schedule Service. Published online December 15, 2021. https://www.va.gov/opal/nac/fss/pharmPrices.asp

14. Heerspink HJL, Stefánsson BV, Correa-Rotter R, et al. Dapagliflozin in Patients with Chronic Kidney Disease. *N Engl J Med*. Published online September 24, 2020:NEJMoa2024816. doi:10.1056/NEJMoa2024816

15. Goldhaber-Fiebert JD, Jalal HJ. Some Health States Are Better Than Others: Using Health State Rank Order to Improve Probabilistic Analyses. *Med Decis Making*. 2016;36(8):927-940. doi:10.1177/0272989X15605091

© 2024 Cusick MM, et al. *JAMA Health Forum*.

16. Richman IB, Fairley M, Jørgensen ME, Schuler A, Owens DK, Goldhaber-Fiebert JD. Cost-effectiveness of Intensive Blood Pressure Management. *JAMA Cardiology*. 2016;1(8):872-879. doi:10.1001/jamacardio.2016.3517

17. Files for FY 2010 Final Rule and Correction Notice | CMS. Accessed June 27, 2022. https://www.cms.gov/Medicare/Medicare-Fee-for-Service-Payment/AcuteInpatientPPS/Acute-Inpatient-Files-for-Download-Items/CMS1247873

18. Vleeming W, van Amsterdam JG, Stricker BH, de Wildt DJ. ACE inhibitor-induced angioedema. Incidence, prevention and management. *Drug Saf.* 1998;18(3):171-188. doi:10.2165/00002018-199818030-00003

19. Sullivan PW, Ghushchyan VH. EQ-5D Scores for Diabetes-Related Comorbidities. *Value in Health*. 2016;19(8):1002-1008. doi:10.1016/j.jval.2016.05.018

20. Cost-Effectiveness Analysis of Canagliflozin 300 mg Versus Dapagliflozin 10 mg Added to Metformin in Patients with Type 2 Diabetes in the United States - PMC. Accessed January 20, 2023. https://www-ncbi-nlm-nih-gov.stanford.idm.oclc.org/pmc/articles/PMC6104269/

21. Clar C, Gill JA, Court R, Waugh N. Systematic review of SGLT2 receptor inhibitors in dual or triple therapy in type 2 diabetes. *BMJ Open*. 2012;2(5):e001007. doi:10.1136/bmjopen-2012-001007

22. Peasgood T, Brennan A, Mansell P, Elliott J, Basarir H, Kruger J. The Impact of Diabetes-Related Complications on Preference-Based Measures of Health-Related Quality of Life in Adults with Type I Diabetes. *Med Decis Making*. 2016;36(8):1020-1033. doi:10.1177/0272989X16658660

23. Recurrent DKA results in high societal costs – a retrospective study identifying social predictors of recurrence for potential future intervention | Clinical Diabetes and Endocrinology | Full Text. Accessed January 20, 2023. https://clindiabetesendo.biomedcentral.com/articles/10.1186/s40842-021-00127-6

24. Sodium–Glucose Cotransporter-2 Inhibitors and the Risk for Diabetic Ketoacidosis: A Multicenter Cohort Study: Annals of Internal Medicine: Vol 173, No 6. Accessed January 20, 2023. https://www-acpjournals-org.laneproxy.stanford.edu/doi/10.7326/M20-0289?url_ver=Z39.88-2003&rfr_id=ori:rid:crossref.org&rfr_dat=cr_pub%20%200pubmed

25. Centers for Disease Control and Prevention (CDC). National Center for Health Statistics (NCHS). *National Health and Nutrition Examination Survey Data*. U.S. Department of Health and Human Services, Centers for Disease Control and Prevention; 2019. https://www.cdc.gov/nchs/nhanes/index.htm

26. Go AS, Chertow GM, Fan D, McCulloch CE, Hsu C yuan. Chronic Kidney Disease and the Risks of Death, Cardiovascular Events, and Hospitalization. *N Engl J Med*. 2004;351(13):1296-1305. doi:10.1056/NEJMoa041031

27. Cooper JT, Lloyd A, Sanchez JJG, Sörstadius E, Briggs A, McFarlane P. Health related quality of life utility weights for economic evaluation through different stages of chronic kidney disease: a systematic literature review. *Health Qual Life Outcomes*. 2020;18(1):310. doi:10.1186/s12955-020-01559-x

28. Nichols GA, Ustyugova A, Déruaz-Luyet A, O'Keeffe-Rosetti M, Brodovicz KG. Health Care Costs by Type of Expenditure across eGFR Stages among Patients with and without Diabetes, Cardiovascular Disease, and Heart Failure. *Journal of the American Society of Nephrology*. 2020;31(7):1594. doi:10.1681/ASN.2019121308

29. Agency for Healthcare Research and Quality. *Number of People in Thousands, United States, 1996-2018.* Agency for Healthcare Research and Quality Accessed November 17, 2020. https://meps.ahrq.gov/mepstrends/hc_use/

© 2024 Cusick MM, et al. *JAMA Health Forum*.

© 2024 Cusick MM, et al. *JAMA Health Forum*.
