# Supplementary material for: When to Start Population-Wide Screening for Chronic Kidney Disease: A Cost-Effectiveness Analysis
Source: JAMA Health Forum. 2024 Nov 8;5(11):e243892. doi: 10.1001/jamahealthforum.2024.3892 (PMC11549659; doi:10.1001/jamahealthforum.2024.3892)
Supplement: Supplement 2. — Data Sharing Statement [file jamahealthforum-e243892-s002.pdf]

Data Sharing Statement

Cusick. When to Start Population-Wide Screening For Chronic Kidney Disease. *JAMA Health Forum*. Published November 08, 2024. doi:10.1001/jamahealthforum.2024.3892

Data
Data available: No

Additional Information
Explanation for why data not available: Data available: No Code available: https://github.com/marikamaecusick/CKDScreeningCEA Explanation for why data not available: No individual patient data were used in this study. Our model was informed by data that are available from published literature and publicly available data sources cited in our manuscript.
